# Recent development of surface-enhanced Raman scattering for biosensing

**DOI:** 10.1186/s12951-023-01890-7

**Published:** 2023-05-06

**Authors:** Chenglong Lin, Yanyan Li, Yusi Peng, Shuai Zhao, Meimei Xu, Lingxia Zhang, Zhengren Huang, Jianlin Shi, Yong Yang

**Affiliations:** 1 State Key Laboratory of High-Performance Ceramics and Superfine Microstructures, Shanghai Institute of Ceramics, Chinese Academy of Sciences, 1295 Dingxi Road, Shanghai, 200050 People’s Republic of China; 2 Graduate School of the Chinese Academy of Sciences, No.19(A) Yuquan Road, Beijing, 100049 People’s Republic of China; 3 Center of Materials Science and Optoelectronics Engineering, University of Chinese Academy of Sciences, Beijing, 100049 People’s Republic of China

**Keywords:** SERS, SARS-CoV-2, Biomolecular, Tumor, Biological imaging, Machine learning

## Abstract

Surface-Enhanced Raman Scattering (SERS) technology, as a powerful tool to identify molecular species by collecting molecular spectral signals at the single-molecule level, has achieved substantial progresses in the fields of environmental science, medical diagnosis, food safety, and biological analysis. As deepening research is delved into SERS sensing, more and more high-performance or multifunctional SERS substrate materials emerge, which are expected to push Raman sensing into more application fields. Especially in the field of biological analysis, intrinsic and extrinsic SERS sensing schemes have been widely used and explored due to their fast, sensitive and reliable advantages. Herein, recent developments of SERS substrates and their applications in biomolecular detection (SARS-CoV-2 virus, tumor etc.), biological imaging and pesticide detection are summarized. The SERS concepts (including its basic theory and sensing mechanism) and the important strategies (extending from nanomaterials with tunable shapes and nanostructures to surface bio-functionalization by modifying affinity groups or specific biomolecules) for improving SERS biosensing performance are comprehensively discussed. For data analysis and identification, the applications of machine learning methods and software acquisition sources in SERS biosensing and diagnosing are discussed in detail. In conclusion, the challenges and perspectives of SERS biosensing in the future are presented.

## Introduction

Surface-enhanced Raman scattering (SERS) phenomenon was innovatively discovered by Fleischmann et al. in 1974 [1]. Since it was officially named by Creighton [2] and Van Duyne [3] in 1977, SERS has attracted the attention and research of many scholars. Compared with detection techniques such as chromatography and immunoassay, Raman spectroscopy combines the advantages of rapidity, sensitivity, and non-destructiveness, and is versatile for solid, liquid and gas samples. Nowadays Raman technology is of great significance in the fields of environmental science, clinical diagnosis, food safety and virus detection [4–8]. In particular, it has bright application prospects in the field of biological analysis due to its non-invasive character and specificity, such as pesticide residue analysis, virus detection, tissue tumor identification, and even bioimaging [9–13].

Traditional SERS substrate materials are mainly noble metals, which are derived from the unique surface plasmon resonance (SPR) effect of noble metals such as gold, silver, and copper [14–17]. These materials are also the main objects for studying metal SERS substrates. The noble metal substrates possess high SERS activity due to their unique SPR effect with an enhancement factor (EF) that can reach 10^14^ [17, 18]. Thus, the detection of individual molecules can be achieved [19]. Generally, nanoparticles of tens of nanometers are more effective, because suitable particles are needed to produce “gap” to enhance Raman signals. The optimal gap is sub-nanometer proximity or have coalesced to form crevices, [20] while a large particle cannot produce a better gap and the particles are more likely to aggregate. However, noble metal substrates also have some inherent disadvantages, such as high preparation cost, easy oxidation, easy agglomeration, poor stability, and low signal reproducibility [21, 22]. With the development of semiconductor nanomaterials, semiconductor SERS substrates are occupying an increasing proportion of the SERS research. Current semiconductor substrate materials can be broadly classified into conventional semiconductor materials (e.g., ZnO, TiO_2_, etc.) [23, 24], organic semiconductor materials (e.g., DFH-4 T, DH-4 T, etc.) [25], 2D semiconductor materials (e.g., MoS_2_, BP, MXene, etc.) [26–28] and semiconductor quantum materials (e.g., Ta_2_O_5_, CdSe, etc.) [29, 30]. Although the explanation for the enhancement of semiconductor is still controversial, the commonly accepted enhancement mechanism is that the photo-induced charge transfer (PICT) between the SERS substrate and the analyte changes the polarizability of the system, which increases the Raman scattering cross section and thus brings about the enhancement of the Raman signal. Semiconductor substrates are considered to be one of the most promising materials due to their high chemical stability, good biocompatibility and controllable preparation process. Of course, semiconductor materials also have obvious shortcomings, that is, the concentration of free carriers in semiconductors is limited and it is difficult to produce SPR effect like metals. For example, the recognized intrinsic carrier concentration of silicon at 300 K is only 1.5 × 10^10^ cm^−3^. While the carrier concentration of Au currently detected is 10^22^ cm^−3^ [31]. Therefore, the SERS sensitivity of semiconductor substrates is generally low. However, no matter noble metals or semiconductors, they can play their respective advantages in specific application scenarios to achieve good results.

In general, there are two SERS methods for biomedical applications, which can be divided into labeling method and unlabeled method [32–34]. The unlabeled method is to obtain the chemical bond vibration information of biomolecules through the direct interaction of the sample with the substrate nanostructure, thus providing the inherent fingerprint information of samples. It is easy to operate, but the signal may be interfered by impurities [35, 36]. The labeling method usually uses Raman reporter molecules with strong and clear Raman signals as SERS tags, which has the advantages of high accuracy and semi-quantitative analysis, but is cumbersome to operate [37, 38]. Designing sensitive and rational plasmonic nanostructures for SERS is essential for successful application of labeling and unlabeled method in biomedical analysis. At present, the SERS sensitivity has been achieved single-molecule detection, which is basically limited to dimers of noble metals or tip-enhanced Raman spectroscopy (TERS) [17, 19]. In addition, it is of course possible to reduce the limit of detection (LOD) of micromolecules to the femto-molar or atto-molar level by some special effects [39, 40]. However, for macromolecules such as amino acids, proteins or cells, especially under the interference of other signals in the environment, realizing the detection of macromolecules still faces great challenges. Although the LOD for probe molecules (e.g. Rhodamine 6G, Crystal Violet, etc.) can be reduced to the level of femto-molar or atto-molar, the LOD decreases to varying degrees when applied to macromolecules detection. This is due to the lack of intrinsic SERS enhancement of traditional SERS templates, the weak adsorption of macromolecules to substrates and the low scattering cross section of macromolecules themselves [21].

**Fig. 1:**
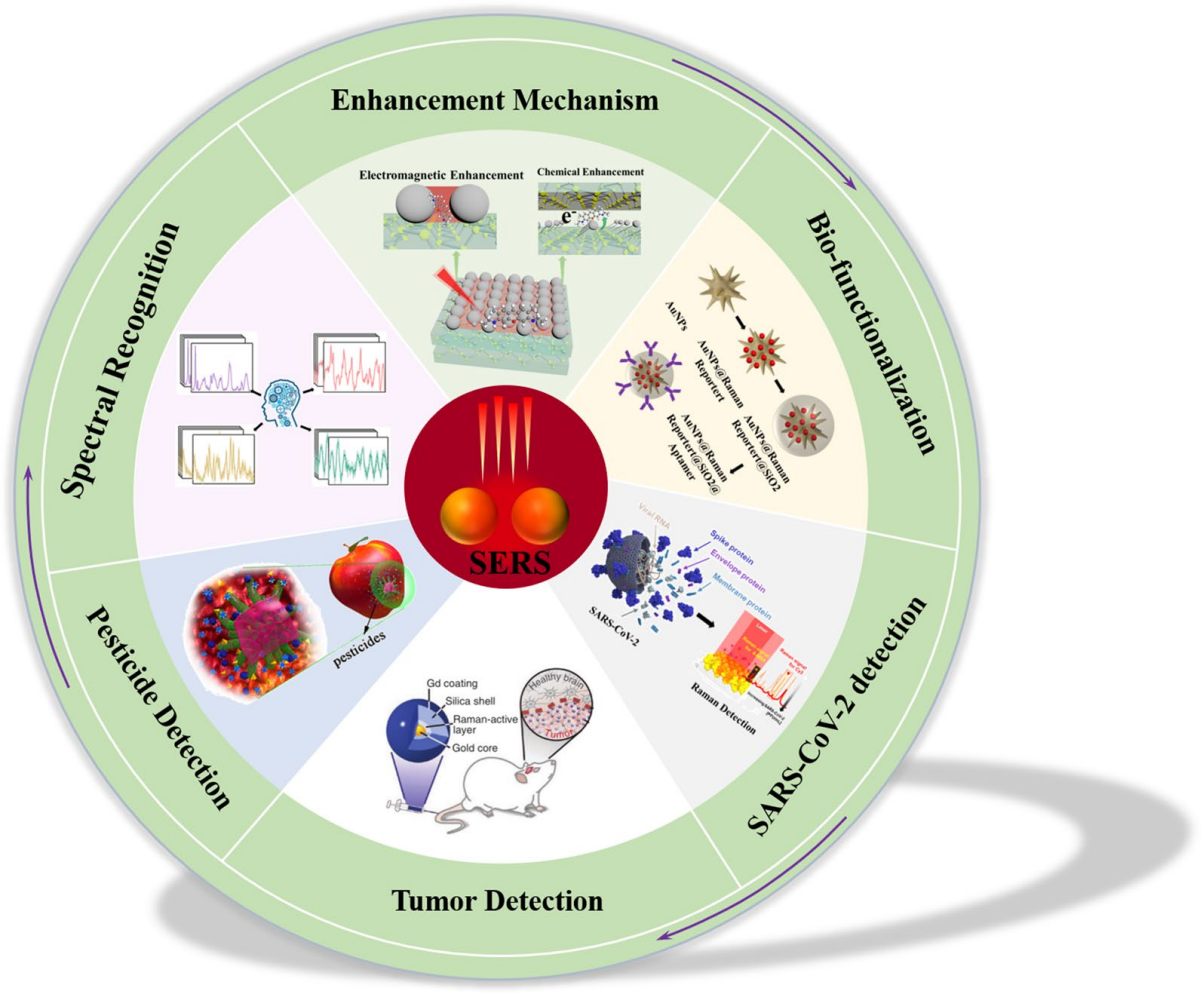
Schematic illustration for various SERS-based applications in biomolecular detection

Therefore, SERS substrates for biomolecule detection usually have the following characteristics: (1) the excellent SERS sensitivity and proper nanostructure design, most of which are noble metals; (2) the excellent biocompatibility; (3) the nanostructure can target binding to analytes; (4) labeling method also requires the reporter molecule with unique and strong Raman fingerprints for labeling method. In the following chapters, we will conduct a detailed review and discussion around the above points. The article structure is given in Fig. 1. In this review, we first briefly discuss the enhancement mechanism of SERS and review the current design and performance of different SERS substrates. Then the strategies of biomolecular capture and detection are summarized and categorized. Subsequently, the application of SERS in SARS-CoV-2 virus detection, tumor diagnosis, biological imaging and pesticide detection is focused on. In addition, reliable spectral identification techniques are an integral part in SERS applications. Finally, promising future trends and prospects are also discussed.

## SERS: Basic theory and progress

In surface-enhanced Raman scattering, molecules are attached to the "nanostructure" of substrates and the Raman signal can be enhanced. This effect was discovered from the pyridine adsorbed on electrochemically rough silver electrodes [1]. In 1979, the enhanced signal of pyridine was also observed in a colloidal solution of silver and gold [3]. Subsequent experiments have shown that SERS is more a “nanostructure effect” than a “surface effect” and clearly demonstrated the important role of surface plasmon resonance in SERS, which corresponds to the electromagnetic mechanism (EM) model developed today [18]. Later researchers demonstrated the important role of charge transfer between the molecule and the substrate in SERS based on the potential distribution measured in an electrochemical environment, which corresponds to the chemical enhancement mechanism (CM) model [41, 42]. These two mechanisms correspond to two models: electromagnetic field enhancement and molecular polarization enhancement. The former focuses on enhanced electromagnetic fields on metal a surface with a suitable morphology, while the latter focuses on the change of the molecular electronic structure during the adsorption process, resulting in resonant Raman scattering. Until 2008, Lombardi et al. gave a unified expression for SERS that integrates surface plasmon resonance, substrate-molecule charge-transfer resonance at the Fermi energy, and an allowed molecular resonance [43, 44].

### Electromagnetic enhancement

Surface roughness or a certain curvature of the substrate is required for the surface plasmons resonance (SPR). The incident laser field and the scattered Raman field can be amplified by the interaction with the surface under the condition of surface plasmon excitation, constituting the electromagnetic SERS mechanism. A good summary of the development of the electromagnetic theories of SERS is given by Moskovits and Tian et al. [45]. The example of a metallic sphere in an external electric field can well illustrate the basic physics of the electromagnetic mechanism [46]. For a spherical particle with a radius much smaller than the wavelength of light, the electric field of the entire particle can be regarded as uniform and the electrostatic approximation can be used. The rough metal surface is similar to the surface of metal spherical particles and the free electrons on the surface of the sphere resemble plasma with unique natural vibration frequency. An excited sphere (radius = r) can be viewed as a dipole and the generated electric field intensity at a distance d from the surface is

1 $${E}_{sp}={r}^{3}\frac{\varepsilon \left(\omega \right)-{\varepsilon }_{0}}{\varepsilon \left(\omega \right)+2{\varepsilon }_{0}} \frac{1}{{(r+d)}^{3}}{E}_{0}$$

where $\varepsilon \left(\omega \right)$ is the complex dielectric constant of the metal and ${\varepsilon }_{0}$ is the dielectric constant of the surrounding environment and ${E}_{0}$ is the electric field intensity of incident light. Therefore, at the distance of d from the spherical surface, the electric field intensity E of the adsorbed molecule can be given as follow:

2 $$E={E}_{0}+{E}_{sp}=\left({1+r}^{3}\frac{\varepsilon \left(\omega \right)-{\varepsilon }_{0}}{\varepsilon \left(\omega \right)+2{\varepsilon }_{0}} \frac{1}{{(r+d)}^{3}}\right){E}_{0}$$

The incident light resonates with the dipole and the electric field intensity of the molecule will be greatly enhanced at the frequency for which $\varepsilon \left(\omega \right)=-2{\varepsilon }_{0}$. The SERS EF is proportional to the fourth power of the local field enhancement factor ($EF\propto {\left|E\right|}^{4}$) so that the Raman signal of the molecule is enhanced on the surface of the sphere [47]. The specific physical model and derivation process can be found in the article of Moskovits et al., and one needs to use the approximation carefully under some circumstances [45]. Generally, such enhancement is realized at the gaps or junctions of plasmonic nanostructures. For example, reducing the gap size in a Au nanosphere dimer from 10 to 2 nm, increases the SERS EF from 10^5^ to 10^9^ [45].

### Chemical enhancement

The EM model does not require a specific chemical bond between the adsorbate and the substrate and can non-selectively amplify the Raman signal of all molecules adsorbed on a specific surface. It explains the enhancement observed at a certain distance from the metal surface. However, under the same experimental conditions, the SERS intensity of molecules CO and N2 differs by a factor of 200 [46]. This result is difficult to explain by EM model alone. The polarizability of the molecules is almost the same, and even the most fundamental difference in orientation during adsorption will not produce such a big difference. The observed anomalies were usually explained using a resonance Raman mechanism in which the charge transfer theory is finally supported. Accordingly chemical enhancement is induced by resonance Raman effect of the charge transfer complex composed of the molecules and metal substrate [48]. Lombardi et al. carried out a comprehensive development of the charge transfer theory of SERS [49]. The theory is comprehensive, considering both molecule-to-metal and metal-to-molecule charge transfers. In addition, the contribution of Franck–Condon and Herzberg-Teller (both theoretically contributed to CT enhancement) to light intensity was also obtained. For noble metal SERS substrates, electromagnetic enhancement plays a major role and chemical enhancement only contributes a small part, while for semiconductor SERS substrates, chemical enhancement usually dominates [42, 50].

Since 1980s, a series of SERS materials such as GaP [51], CdTe [52] and TiO_2_ [24] have been discovered, and extending CT resonance to the plasmon-free SERS materials. It should be noted that CT complex is formed by the strong chemical bonding between the molecule and semiconductor. In the semiconductor-molecule system, the CT process would strongly depend on the efficiency of the electronic coupling between the conduction band (CB) and valence band (VB) of semiconductors with the highest occupied molecular orbital (HOMO) and the lowest unoccupied molecular orbital (LUMO) of the adsorbed molecules. In addition, some plasmon-free metallic materials also show ultrasensitive SERS sensing capacity, which involves in the CT process between the energy level of molecules and the Fermi level of metallic materials [53].

### A unified expression for SERS

The theory of SERS enhancement introduced above is constantly improving. At present, it is generally believed that there are three possible contributions to enhancement factors: (1) plasma resonance on the surface of metal nanoparticles, (2) charge transfer resonance between molecules and substrates, and (3) resonance inside the molecule. These three parts are usually considered as independent contributions to the EF, which means that one or more can be ignored by correctly selecting the experimental parameters. Although different experimental conditions will affect each resonance, higher enhancement can usually be obtained by combining multiple resonances. Each resonance has a somewhat effect on the Raman spectrum and it is necessary to quote one or more of these resonances to fully describe Raman enhancement. If these three contributions are not considered simultaneously, it is impossible to completely describe all the observations of SERS phenomenon. Accordingly Lombardi et al. gave a unified expression of SERS enhancement, and proved that the three resonances are closely related by the Herzberg-Teller vibration coupling term and cannot be considered separately [43]. In this formula, the polarization tensor is

3 $${R}_{IFK}\left(\omega \right)=\frac{{\mu }_{KI}{\mu }_{FK}{h}_{IF}<i\left|{Q}_{K}\right|f>}{({\left({\varepsilon }_{1}\left(\omega \right)+2{\varepsilon }_{0}\right)}^{2}+{{\varepsilon }_{2}}^{2})({{\omega }_{FK}}^{2}-{\omega }^{2}+{{\gamma }_{FK}}^{2})({{\omega }_{IK}}^{2}-{\omega }^{2}+{{\gamma }_{IK}}^{2})}$$

Raman intensity is proportional to the square of polarizability, i.e., ${\left|{R}_{IFK}\left(\omega \right)\right|}^{2}$. I, F and K in the expression represent the ground state, charge transfer state and excited molecular state of the molecular-metal system, respectively. The numerator in formula (3) provides the selection rules for SERS, and all four terms are interrelated. The Herzberg-Teller effect contributes a product of ${h}_{IF}=\langle I\left|\partial {V}_{eN}/\partial {Q}_{k}\right|F\rangle$ with $<i\left|{Q}_{K}\right|f>$. The other two terms in the numerator relate to the dipole transition moment ${{\mu }^{\sigma }}_{KI}{{\mu }^{\rho }}_{FK}$, which are the allowed molecular transition I-K and metal molecular charge transfer transition F-K. For denominator, the first term in the denominator represents plasma resonance at ${\varepsilon }_{1}\left(\omega \right)=-2{\varepsilon }_{0}$, where ${\varepsilon }_{1}$ and ${\varepsilon }_{2}$ is the real part and imaginary part of the dielectric constant of the metal and ${\varepsilon }_{0}$ is the real part of the dielectric constant of the surrounding medium. Here, a single spherical particle is taken as an example, while for other non-spherical hot spot structures, a similar but more complex expression including dielectric resonance is required [54]. The second resonance is potential dependent and represents charge transfer resonance at ${\omega }_{FK}=\omega$. The third resonance is molecular resonance at ${\omega }_{IK}=\omega$.

Therefore, the denominator of Eq. (3) can predict the possibility of these resonances simultaneously, which depends on the metal and molecular parameters. If the contribution of each resonance is equal (depending on the damping parameters ${\varepsilon }_{0}$, ${{\gamma }_{FK}}^{2}$ and ${{\gamma }_{IK}}^{2}$), it can be predicted the enhancement of 10^3^–10^4^ for one resonance, 10^6^–10^8^ for two resonances and 10^9^–10^12^ for all three resonances. Of course, all three damping parameters are usually not equal and each item will contribute to different degrees in the actual experiment at any given excitation wavelength. In addition, for any resonance the enhancement factor is proportional to the inverse fourth power of the corresponding damping parameter (${\gamma }^{-4}$, $\gamma$ is ${\varepsilon }_{0}$、${\gamma }_{FK}$ or ${\gamma }_{IK}$). Therefore, the magnitude of SERS enhancement is very sensitive to the magnitude of these parameters. In order to obtain better SERS enhancement, it is not possible to consider a single resonance, which is the reason why many materials cannot achieve ideal enhancement even under resonance conditions.

In addition to the above-mentioned electromagnetic resonance, CT resonance and molecular resonance, Mie resonance also has an important effect on SERS enhancement. Compared with localized surface plasmon resonance (LSPR) in metallic particles, Mie resonance typically exists in dielectric particles [55]. It is related to the particle size and incident wavelength. Mie scattering causes Raman enhancement when the particle size is comparable to the wavelength of incident light. This situation can be identified by an important size parameter χ (2πr/λ), where r is the radius of the spherical particle and λ is the wave number of the incident light. The Mie system is defined as 0.1 < χ < 100. As early as 1988, Hayashi et al. observed the Raman enhancement of copper phthalocyanine (CuPc) molecules adsorbed on GaP nanoparticles of different sizes and explained the Raman enhancement in plasmon-free materials by Mie scattering theory for the first time [51]. Alessandri et al. observed obvious Raman enhancement on TiO_2_ shell-based spherical resonator, which can be explained by multiple light scattering through the sphere, high refractive index of the shell layer, and related geometrical factors [56]. They also demonstrated that Mie resonances can be generated in SiO_2_/ZrO_2_ core/shell beads of 2 μm size [57]. Ji et al. also found a significant enhancement of Raman sensitivity on submicron spherical ZnO, which was attributed to the synergistic effect of CT in ZnO nanocrystals and the Mie resonance of the superstructure [58]. Furthermore, numerous studies have shown that plasma-free meta-SERS strongly depends on material size, surface defects, sample morphology, crystallinity, and crystal orientation [55]. Whereas most of these factors can be correlated with the previously mentioned electromagnetic resonance, CT resonance or Mie scattering.

## Bio-functionalization engineering for SERS biosensing

The utility and versatility of SERS has aroused wide acceptance in the biomedical analysis. In order to obtain highly sensitive, accurate, and fast biosensors, continuous improvements have been made to SERS substrates. Several functional groups can be immobilized on the surface of templates for the surface-dependence of substrate, such as the selection of SERS active materials using localized surface plasmon resonance, design of surface structures, and modification of affinity agents. In particular, affinity agents not only confer high selectivity to the substrate, but also concentrate the targets on the substrate surface to improve sensitivity. This section focuses on surface modifications, including antibodies, aptamers, and polypeptides.

### Functionalization techniques based on antigen/antibody

Antibodies are immune proteins expressed in B cells and contain molecular recognition sites that specifically bind to their targets. Although the antibodies have the same structure conformation, the binding region is various for different antigen. The binding between antibody and antigen is due to hydrophobic interaction, hydrogen bonds, van der Waals forces, and ionic bonds [59]. Due to the lack of specificity and selectivity, traditional SERS substrates are not suitable for practical detection in complex matrices. Inspired by immunoassay, specific antibodies can be functionalized on the surface of substrate to selectively capture the target. Functionalized SERS substrates can eliminate the interference of other component in matrix and provide high sensitivity and specificity. Antibody-functionalized SERS biosensors are usually divided into two types: direct and indirect methods. Direct SERS sensors consist of SERS-active materials, antibodies and antigens. Unlike the direct detection, indirect method always consists of SERS-active materials, antibody, antigen, and probe molecules.

Comparing the indirect method, direct method can accurately acquire the molecule information of targets. Myeong-Lok Seol et al. fabricated a label-free SERS immunosensor with nanoforest structure for detecting influenza A virus subtype H1N1, which is based on the SERS signal differences caused by the selective binding of the H1N1 surface antigen and the anti-H1 antibody [60]. He et al. presented a novel SERS biosensors combined immunomagnetic separation (IMS) for detecting ovalbumin (OVA) in milk [61]. The IMS eluate was analyzed based on SERS spectra using Ag dendrites as substrate directly. Maria Knauer et al. developed a new technique for label-free microarray readout based on SERS [62]. The technique is not only able to detect microorganisms in an aqueous environment in-situ, but also achieves the nondestructive analysis of living bacteria cells.

**Fig. 2:**
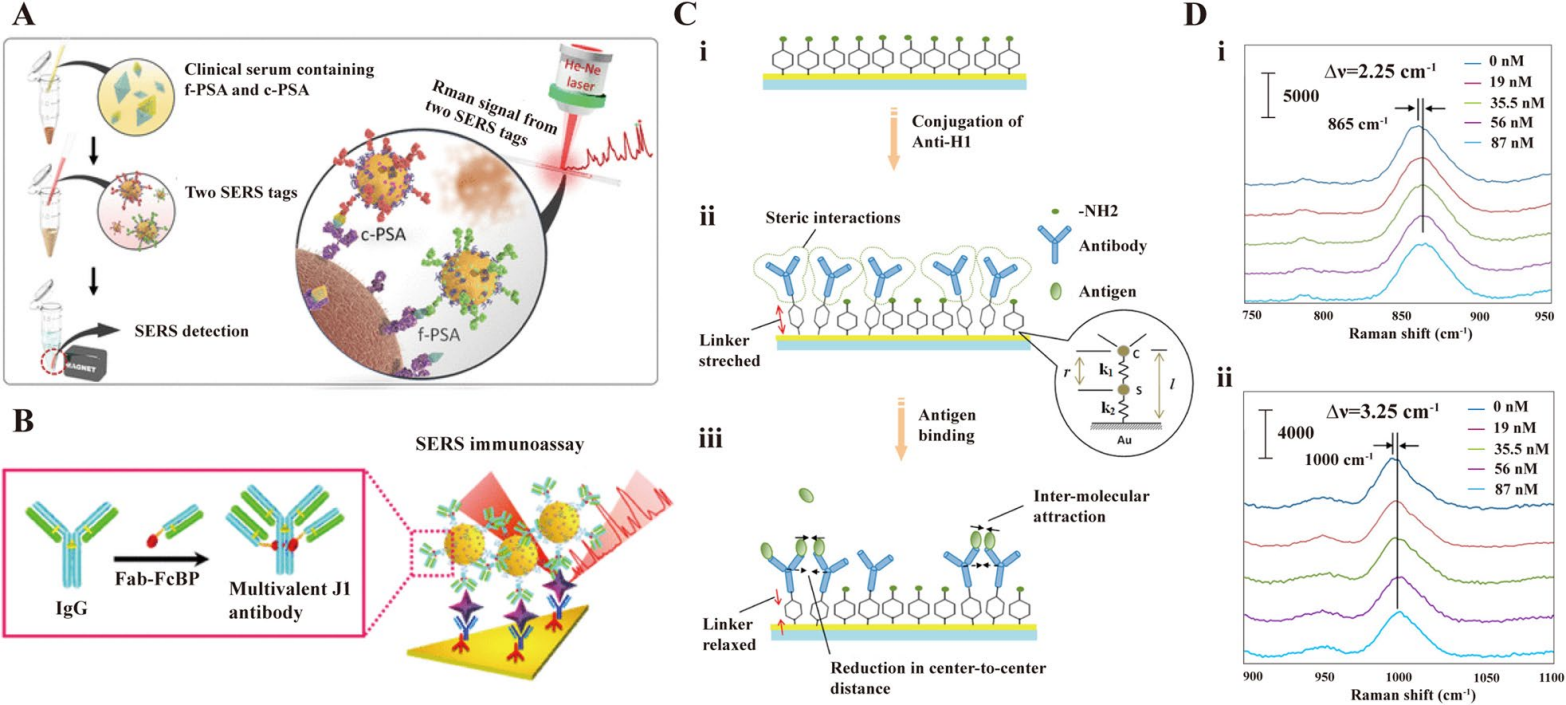
SERS biosensor based on antibody/antigen. **A** Sequential SERS-based assay process for the simultaneous detection of f-PSA and c-PSA. [63] **B** Schematic illustration of MANC preparation for J1 peptide detection. In the presence of J1, MANC-on-nanoplate structures are constructed and SERS signals of MGITC are observed. [67] **C** Mechanical deformation in an anti-H1/4-ATP sensor. (i) A pure 4-ATP SAM, (ii) Conjugation of anti-H1 leads to stretching of the 4-ATP molecule, (iii) Binding of H1 leads to reductions in the center-to-center distance between antibody molecules, thereby leading to mechanical relaxation in 4-ATP. [68] **D** Average SERS spectra at different antigen concentrations, showing shifts at (i) 865 and (ii) 1000 cm^−1^. [68] **A** reprinted with permission from Ref. 63, © 2017, American Chemical Society. **B** reprinted with permission from Ref. 67, © 2018, American Chemical Society. **C** and **D** reprinted with permission from Ref. 68, © 2012, American Chemical Society

The label-free SERS detection faces a grant limitation which is the poor spectral reproducibility. Therefore, indirect detection is another alternative road. Cheng et al. developed SERS-based immunoassay consisted of magnetic beads and SERS nanotags for the determination of free to total (f/t) prostate specific antigen (PSA) ratio (Fig. 2A) [63]. The diagnostic methods could achieve the simultaneous detection of dual PSA biomarkers within the gray zone between 4.0 and 10.0 ng/mL for clinical samples. Jiang et al. constructed an Fe3O4@TiO_2_@Au nanocomposite immunoprobe with a detection limit of 1.871 pg/mL LOD for PSA [64]. Notably, the SERS immunoprobe can be recycled multiple times due to the excellent catalytic properties of TiO_2_. The functionalized SERS-based assay method with special antibody was modified further to enhance the sensitivity and selectivity. In biosensing, the use of multivalency can be highly advantageous because the increased valency can increase the binding affinity between the receptor and target molecules up to tens of times, improving the biosensor sensitivity [65, 66]. Miyeon Lee et al. reported a SERS-based immunoassays using multivalent antibody-conjugated nanoparticles (MANCs), which could improve the sensitivity of biosensors because of the increasing binding affinity between the receptor and targets [67]. As depicted in Fig. 2B, multivalent antibodies were constructed with immunoglobulin G linked by Fab fragments fused with Fc-binding peptides. Comparing the standard antibody-NP conjugates, the MANCs could improve the sensitivity of SERS-based assay by 100 times. Kiang Wei Kho et al. reported a novel SERS sensor based on the vibrational frequencies of antibody-conjugated SERS-active reporter complex [68]. The Raman shift of stress-sensitive SERS reporters will slightly change when the antigen is present, the SERS-active nanomechanical sensor is applied to detected biomolecules with high-sensitivity based on above guideline (Fig. 2C). Upon binding to the antigen, the steric repulsion could be overcome by the attractive hydrophobic interactions between the bound antigens. This results in a slightly closer of the antigen–antibody complexes and relaxing the tensile deformation within the 4-ATP structure, which in turn upshifts both the 865 and 1000 cm^−1^ peaks (Fig. 2D).

SERS biosensors modified with antibody is based on the high affinity and interaction between antibody and targets. The design of different antibodies on the SERS substrate can be used for the capture, isolation, and detection of multiple biomarkers. Excellent sensitivity of SERS detection could be acquired by selectively identifying and binding targets, which has enormous potential in the detection of biomolecules, metal ions, food additives, etc [69].

### Functionalization techniques based on aptamer

Aptamers are short single-strand DNAs or RNAs, which possess high affinity and specificity for diverse targets, including metal ions, organic molecules, biomolecules, and microorganisms/cells. The structure deformation of aptamer around the target promoting intermolecular interactions, taking advantage of van der Waals forces, hydrogen bonding, and electrostatic interactions to form a stable target-aptamer complex [70]. Compared with antibodies in bioanalysis, aptamers is easier to synthesize with high performance/cost ratio and ultrahigh binding affinity to targets [71, 72]. Moreover, aptamers are more stable than antibodies because of avoiding biodegradation and denaturation.

**Fig. 3:**
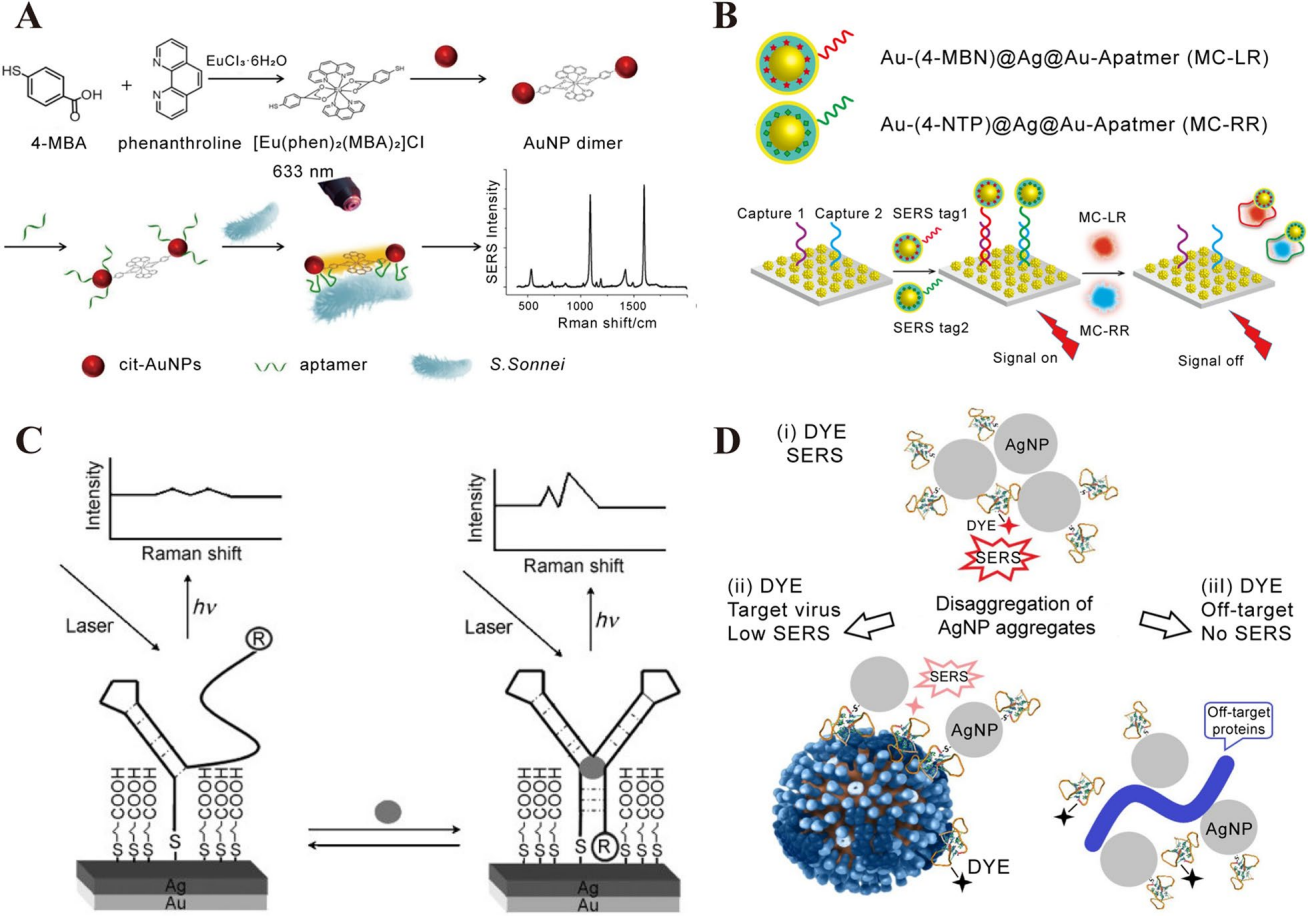
SERS biosensor based on aptamer. **A** Schematic representation of SERS aptasensor for S. Sonnei determination utilizing dual-functional metal complex-ligated gold nanoparticles dimer. [74] **B** (i) Sequential procedure for fabricating two types of aptamer-modified NP SERS tags. (ii) Procedure for fabricating a dual MC sensor for MC-LR and MC-RR. [75] **C** Schematic diagram for the preparation and analytical principle of the aptameric sensor for cocaine. [76] **D** Schematic representation of aptasensor setup. Aptamer-functionalized AgNP were mixed with a labeled aptamer in buffered saline providing AgNP aggregates (ii). The aggregates were mixed with target viruses (iii) resulting in weaker SERS signals or with off-target biologicals (iv) losing SERS effect due to the elimination of the labeled aptamer from AgNP aggregates. [77] **A** reprinted with permission from Ref. 74, © 2020, Elsevier. **B** reprinted with permission from Ref. 75, © 2021, American Chemical Society. **C** reprinted with permission from Ref. 76, © 2008, John Wiley and Sons. **D** reprinted with permission from Ref. 77, © 2021, International Journal of Molecular Sciences

The distance and absorption strength between probe and SERS substrate would significantly affect the Raman intensity. Based on the guideline, aptamer could be designed to enhance the SERS performance [73]. The binding-induced conformational change of aptamer would cause the change of Raman intensity, which can be the principle to design SERS aptasensor. Wu et al. constructed a SERS aptasensor for the sensitive and specific detection of Shigella sonnei with the LOD of 10 cfu/mL [74]. With the introduction of S. Sonnei, the binding-induced deformation of aptamer could immobilize the bacteria in close proximity of Au NPs (Fig. 3A). Luo et al. developed a SERS aptasensors combined the Au@label@Ag@Au nanocomposite functionalized with MC-LR and/or MC-RR (two species of microcystins family) aptamers and Au nanoflowers (Fig. 3B). The SERS aptasensors could indirectly detect Microcystins (MCs) in natural water and algal culture with the LOD of 0.8 pM for separate MC-LR detection [75]. After inducing MC-RR and MC-LR, the corresponding aptamers would deform and dissociate from the Au nanoflowers because of the high affinity between aptamers and MCs. Raman signal would significantly decrease due to the removement of nanocomposite functionalized aptamers. Chen et al. reported aptameric SERS sensor to detect cocaine [76]. With the present of cocaine, the TMR-labeled DNA aptamer was in close to the SERS substrate because of the conformational change, and the optical enhancement could be increased significantly (Fig. 3C).

A favorable aggregation is caused by aptamer, which is in favor of the formation of “hot-spot” and increases the optical enhancement. Dmitry Gribanyov et al. developed a kind of SERS aptamers for quantitative determination of influenza virus based on the aptamers-induced aggregation of AgNPs [77]. As shown in Fig. 3D, aptamer-induced aggregation of Ag-NPs will enhance the SERS intensity. However, the existence of virus in colloidal solution leads to the disaggregation of AgNPs and the SERS intensity turns weaker or disappears. Wu et al. described a novel aptamer sensor based on AuNPs tetramers for ultrasensitive determination of the agent oxytetracycline (OTC) [78]. The sensing principle is due to the aggregation and disaggregation of Au NPs tetramers. In the absence of targets (OTC), strong Raman intensity can be acquired because of the gap between adjacent AuNPs. However, the disaggregation will take place and the Raman intensity will decrease, which is due to the binding between aptamer and targets (OTC). Some other 2D semiconductor materials, such as MoS_2_, MXene, BP and hexagonal boron nitride, are used as SERS active substrates because of their large surface area, ease of functionalization and high loading capacity [79–82]. In general, these semiconductor materials are required to form complexes with metal nanoparticles in order to obtain better SERS performance. The intense electromagnetic effect formed in the gap among deposited nanoparticles and additional chemical enhancement on the 2D surface as well as the high affinity to the analyte may contribute to the superior performance of this type of SERS substrate. Pan et al. designed a MoS_2_-AuNSs nanocomposite and assembled ROX-labeled aptamers on the MoS_2_- AuNSs surface as a recognition probe to detect exosomes. The LOD of this SERS aptasensor exosomes was 17 particles/μL, which is better than many noble metal particle-based SERS aptasensors [79]. Liu et al. designed a MXene/MoS2@AuNPs with controllable morphology for the ultrasensitive detection of miRNA-182 and a linear detection window from 10 aM to 1 nM with an ultralow detection limit of 6.61 aM is achieved [80].

SERS aptasensor is a kind of promising sensor with high-sensitivity, specificity, and accuracy, which possesses tremendous potential for multiplex detection. The key technical problems to be solved in application are produce uniform SERS substrate and reproducible SERS assays.

### Functionalization techniques based on polypeptide

**Fig. 4:**
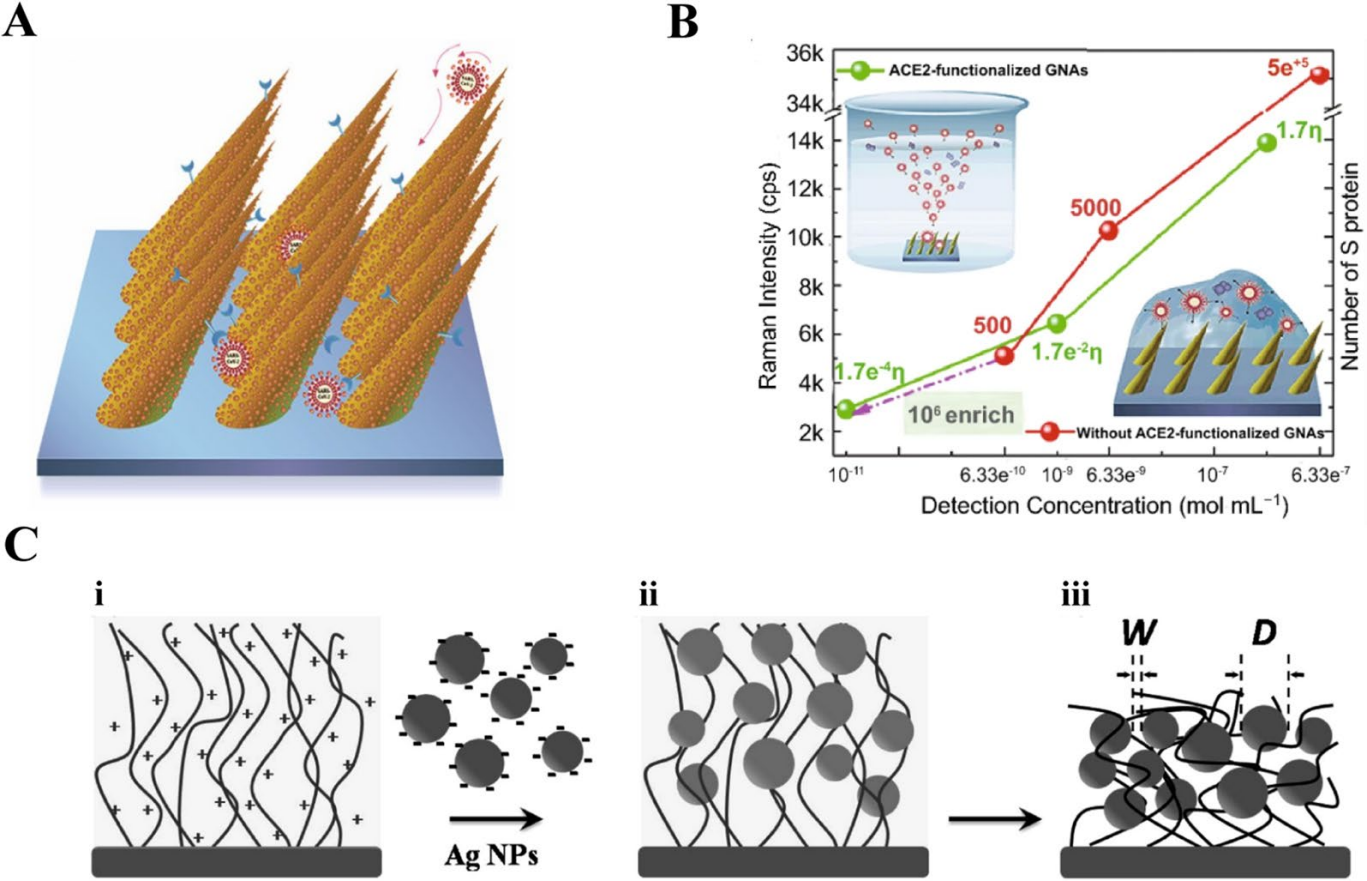
SERS biosensor based on peptide. **A** Schematic diagram of “virus traps” nanostructure SERS sensor for capturing SARS-CoV-2. [6] **B** Intensity of Raman bands (1027 cm^−1^) of SARS-CoV-2 S protein with different concentration detected with ACE2 functionalized GNAs and without ACE2 functionalized GNAs. The value marked on the line represents the number of S proteins in one Raman focused window. η represents enrichment multiple by ACE2. [6] **C** Schematic illustration of the fabrication of Ag NP-t-PLL film. (i) The amine groups of PLL chains of the t-PLL brush exposed positive charges in Ag NPs solution. The negatively charged Ag NPs were conjugated onto the film via strong electrostatic interaction and thus the (ii) Ag NP-t-PLL film in solution was formed. The film was removed from Ag NPs solution and then washed by deionized water. After the film was dried, (iii) the Ag NP-t-PLL film was prepared, and the W and D of Ag NP-t-PLL film were also defined. [90] **A** and **B** reprinted with permission from Ref. 6, © 2021, Springer Nature. **C** reprinted with permission from Ref. 90, © 2009, American Chemical Society

Polypeptide is a compound consisting of various amino acids bound by peptide bonds in a certain order [83]. High stability, lost-production, easy functionalization, and affinity with targets make polypeptide an important part of biosensor design [84]. A number of specific polypeptides have been used to recognize and enrich targets on the surface of SERS substrates. The function of polypeptides on SERS-active substrates is similar with that of antibody modified, which aims to capture more targets and improve the SERS enhancement. Xie et al. developed a novel nuclear targeting nanoprobe based on Au NPs functionalized specified peptide for the direct SERS detection of living cells [85]. Lee et al. used Ag NPs functionalized thiolated peptide ligand to capture exosomes for analyzing the α3β1 integrin over-expressed in exosomes, and the SERS biosensor processed high specificity comparing to the Raman spectra of negative exosomes [86]. Wang et al. reported a king of SERS biosensors for direct diagnosing circulating tumor cells (CTCs) in whole blood, and the epidermal growth factor peptide served as targeting ligand to capturing CTCs [87]. The SERS biosensor successfully identified CTCs with a range of 1 to 720 CTCs per milliliter of whole blood. Our group have developed a kind of Human Angiotensin‑converting‑enzyme 2 (ACE2)‑functionalized gold “virus traps” nanostructure as SERS biosensor for the detection SARS-CoV-2 (Fig. 4A) [6]. The simulated EM enhancement is localized within a dozen nanometers of nanoneedles. ACE2 can specifically capture SARS‑CoV‑2 and localize S protein of virus within the strongest‑SERS area of 10 nm, leading to high‑enhanced Raman signals of S protein. As shown in Fig. 4B, the nanostructures showed LODs of 0.63 nM and 17.7 pM for S protein before and after modifying ACE2, respectively. The SERS biosensor possessed 10^6^-fold enrichment because of the high-affinity of ACE2 to S protein and the “virus trap” nanostructure. The introduction of targets will change the conformation of polypeptide modified on the surface of substrates. According to the variety of Raman signal caused by above change, the SERS detection of targets could be achieved. Sun et al. presented a novel SERS biosensor for detecting caspase-3 with Au nanoboxes, Nile blue A as a Raman reporter, and a caspase-3-specified peptide as cross-linker [88]. Excellent SERS performance can be obtained by the aggregated Au nanoboxed which is due to the cleaving peptides and changed surface charge of Au nanoboxes in the presence of caspase-3.

Noble metals are the most ideal SERS substrate, but the direct synthesis of noble metals is liable to aggregate. It is a giant challenge to control the size and shape of noble metal nanoparticles. To obtain the maximized SERS enhancement, it is necessary to adjust the ration (W/D) of nanoparticle diameter (D) and interval width (W). Polypeptides not only could serve as the specific affinity agents to capture targets, but also act as a template to control the synthesis of SERS-active substrates with high sensitivity. [89] Wang et al. fabricated Ag film (Ag NP-t-PLL film) constructed of Ag NPs and biocompatible end-tethered poly (L-lysine) (“t-PLL”) with a brushlike configuration, and the scheme of preparation process was shown in Fig. 4C [90]. The conjugation between Ag NPs and “t-PLL” was adjusted to acquire the most optimal ration (W/D), which increased the SERS enhancement significantly. Moreover, polypeptides could affect the surface state of SERS substrate, which is in favor of the SERS enhancement. Helena Domin et al. discussed the colloidal and electrochemically roughed Ag and Au substrates immobilized on polypeptides [91]. Some change in the surface geometry was observed, which is mainly due to the Tyrosine ring(s) being parallel to the substrates and in the close contact with the substrate surface.

SERS sensors functionalized by polypeptides possesses excellent sensitivity duo to high affinity between targets and substrate. By rational selection and design of the optimal structure or polypeptide, it is possible to obtain the most accurate and sensitive SERS assay.

## Biosensing applications in several important bio-fields

SERS technology has been developed for several decades and the research on mechanism or substrate materials has been well studied. The further development of SERS must be combined with practical application to solve the problems faced in practical testing. In recent years, internal and external SERS biosensors have been widely used to detect and recognize macromolecules, nucleic acids, peptides and proteins, as well as for cellular sensing.

### SARS-CoV-2 detection

The spread and transmission of virus have become a threat to worldwide public health, especially the current pandemic SARS-CoV-2, which has the characteristics of high-speed transmission and rapid production of new variants [92]. Conventional detection methods, such as ELISA or quantitative real-time PCR (RT-qPCR), require specific probe molecules of virus and long reaction time [93, 94]. Facing new viruses’ variants, generous time and professional technicians are required to construct probe molecules of new virus. Other genetic methods, such as gene sequencing, reverse transcription loop mediated isothermal amplification (RT-LAMP) or CRISPR (clustered regular interspaced short palindromic repeats) diagnosis, also have some inherent shortcomings [95–99]. In the past decades, SERS has proved itself to be a highly selective tool in the field of virus diagnosis. SERS sensors have been successfully applied to detect various viruses, such as influenza virus [9, 100], dengue virus (DENV) [101], human immunodeficiency virus (HIV) [102], ebola virus [103], severe acute respiratory syndrome coronavirus (SARS-CoV) [104], etc. Especially since the COVID-19 pandemic, a large number of researchers have rapidly applied SERS sensing to the detection of SARS-CoV-2, and strive to promote its practicality and industrialization, which is the focus of our attention [6, 105–111]. Table 1 showed the advantages/disadvantages of some commonly used genomic analysis, electrochemical sensors, plasma (SPR) sensors, and SERS sensors for virus detection. Compared with other virus diagnosis methods, the most prominent advantage of SERS method is its short detection time.

**Table 1:** Comparisons of the different methods for the detection of subtypes of SARS-CoV-2

Method	Pros	Cons	Refs.
Genome sequencing	High accuracy and reliability, gold standard for identifying variants	Low sensitivity, specialized laboratories and technical skills, be time-consuming and expensive	[95]
RT-qPCR	High sensitivity, reliability	primer/probe mismatches, expensive equipment	[94, 112]
RT-LAMP	High specificity, portability, be rapid and costly	low tolerance to highly variable target sequences, limitations of a single reaction	[96, 97]
CRISPR	High specificity and sensitivity, experimental simplicity, versatility	Shortage of multiplexing capabilities	[98, 99]
ELISA	Fast response, Portability and simplicity	Low sensitivity	[93, 113]
Electrochemical method	High sensitivity, fast response and low cost, small size, and portability	Weak stability and susceptibility to interference	[114, 115]
Magnetic biosensors	low cost, high signal-to-noise ratio test	Bulky equipment	[116, 117]
SPR-based biosensors	Label-free and real-time detection	Bulky equipment and expensive, low sensitivity	[118, 119]
SERS-based biosensors	High sensitivity, Portability and simplicity, low cost	Poor spectra repeatability	[120]

#### Label-free SERS for the detection of SARS-CoV-2

**Fig. 5:**
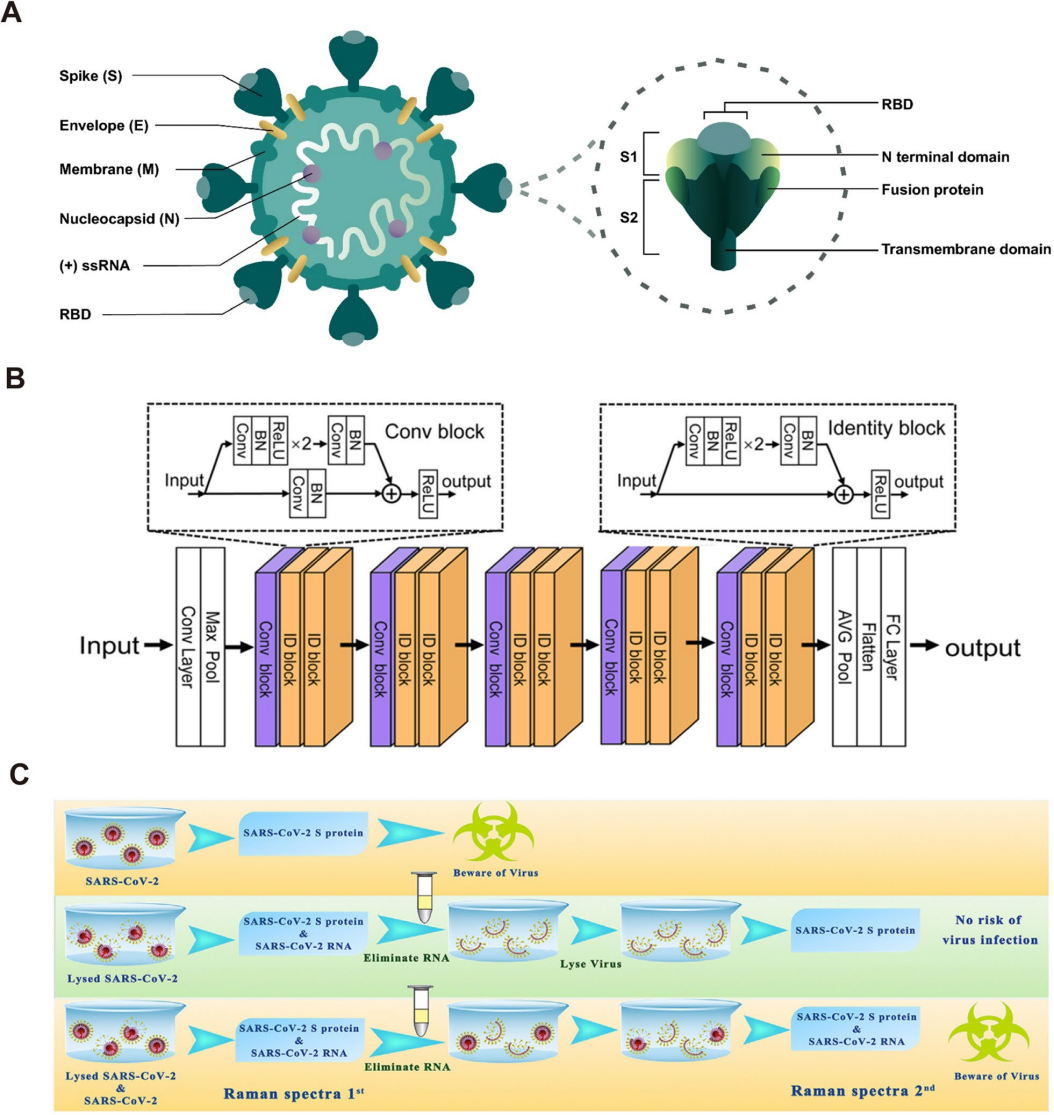
Label-free SERS for the detection of SARS-CoV-2. **A** Schematic representation of SARS‑CoV‑2 and spike glycoprotein main structural features. [122] **B** The framework of the CNN deep learning model for the diagnosis of SARS-CoV-2. [109] **C** Experimental procedure for diagnosing the infectiousness of SARS-CoV-2 [120]. **A** reprinted with permission from Ref. 122, © 2021, Springer Nature. **B** reprinted with permission from Ref. 109, © 2021, American Chemical Society. **C** reprinted with permission from Ref. 120, © 2022, Elsevier

The label-free SERS technique directly acquires the Raman spectra of the substance to be measured. By analyzing the corresponding Raman vibrational spectra, the molecular structure of the substance to be measured can be analyzed. In particular, it can be used for distinguishing different viruses and identifying virus variants. It is not only possible to differentiate different viruses from the perspective of spectral vibrations, but also to further analyze and verify the actual variant properties of virus nucleic acids and proteins. However, the large size of viruses relative to small molecule compounds makes it difficult to obtain standard Raman spectra. The identification of viral Raman spectra is usually based on the Raman signal of certain proteins on the surface of the virus [121]. As shown in Fig. 5A, four main structural proteins, essential for the complete assembly of the viral particle are encoded by the coronaviral genome: the spike S protein, the nucleocapsid N protein, the membrane M protein, and the envelope E protein [122]. Each protein has a specific function: the S protein mediates the adhesion and subsequent fusion between virus and host cell receptor; the N protein binds to the CoV RNA genome, arranges the nucleocapsid, and participates in the viral replication cycle; the M protein forms the major structural part of the viral envelope and interacts with all other structural proteins; and the E protein is the smallest integral membrane structural protein incorporated into the viral envelope, is important for virus production and maturation. Considering the size, quantity and structural characteristics of the proteins, S and N proteins are the easiest targets to use for Raman detection.

The S protein of SARS‑CoV‑2 consists of two subunits: the S1 subunit contains a receptor‑binding domain (RBD) that binds to angiotensin‑converting enzyme 2 (ACE2) on the surface of host cells, whereas the S2 subunit mediates fusion between the membranes of the virus and the host cell. The S protein has a nail-like shape with a width of 7 nm and a length of 23 nm, has the maximum probability to fall into the SERS hot spots [109]. Therefore, the S protein of SARS-CoV-2 is a significant target in SERS sensing. Since the outbreak of COVID-19 in 2019, our research group has been engaged in the detection of SARS-CoV-2 based on SERS sensors, and has completed the rapid detection of SARS-CoV-2 S protein and single virus respectively [6, 28, 111, 123]. We have pioneered the study of the Raman characteristic spectra of SARS-CoV-2 [28]. This is the first report of Nb_2_C material with good SERS activity and accurate identification of the Raman peak of SARS-CoV-2 S protein. This has important implications for the use of Raman technology for real-time monitoring and early warning of SARS-CoV-2. Huang et al. established a Raman database based on the SARS-CoV-2 S protein through experiments and theoretical calculations, and achieved rapid on-site detection of SARS-CoV-2 antigen based on deep learning [109]. As shown in Fig. 5B, this research gives a convolutional neural network (CNN) model capable of being used for Raman spectral classification and recognition, which is able to solve the gradient vanishing problem of deep architecture. The framework of the CNN was organized with an initial convolution layer, followed by batch normalization (BN), a rectified linear (ReLU) transformation, and a max pooling layer (Max Pool), followed by five serial blocks. These blocks were one convolution block followed by two identity blocks with shortcuts. Finally, these blocks were followed by an average pooling layer (AVG Pool), a flatten layer, and a fully connected (FC) layer. A well-prepared dataset is essential to deep learning-based SERS for the diagnosis of SARS-CoV-2. The training set needs to take into account the interference of the spectrum caused by various factors that may occur in the actual detection to improve the accuracy of the model in identifying viruses. Because the method does not have a protein purification process and overly relies on the deep learning model and data volume, the sensitivity and specificity of its recognition cannot yet satisfy the application requirements. Therefore, in practical applications we must consider the purification process of the virus. By designing appropriate nanostructures, the virus can be captured and limited to hot spots through ACE2, which could specifically detect the S protein of the virus. Our group then designed a "nano-forest" SERS chip for the capture and detection of SARS-CoV-2. The ACE2 receptor on this SERS chip confines the virus to the "nanoforest" and specifically enhances the Raman signal of the S protein [6]. The detection time of this method is 5 min, and the LOD can reach 80 copies/ml. Wang et al. also reported an ACE2-modified SERS biosensor to detect SARS-CoV-2 in medical wastewater with an accuracy of 93.33% [124].

Another striking advantage of label-free SERS sensing for detecting surface proteins of virus is that it can estimate the activity (infectivity) of the virus, which cannot be achieved by PCR. As shown in Fig. 5C, our group have provided a scheme that can detect the infectivity of coronavirus for the first time, through comparing the Raman signals with S protein and RNA in the established database [120]. Since no specific primers are required, SERS sensing also has great advantages in dealing with virus variants and frequent mutations. Choi et al. successfully identified several different influenza viruses and shuffled influenza viruses using SERS sensors [9, 100]. Similarly, SERS sensing can also deal with the current outbreak of coronavirus and obtained satisfactory results. To date, seven coronaviruses are known to endanger human health, 229E and NL63 from the α coronavirus genus, and HKU1, OC43, MERS‑CoV, SARS‑CoV and SARS‑CoV‑2 from the β coronavirus genus [125]. Table 2 showed the comparison of different SERS sensors used for coronavirus detection.

**Table 2:** Comparisons of different SERS biosensors for the detection of subtypes of SARS-CoV-2

Method	Analytes	SERS substrate	Detection time	Limitation of detection	Physiological environment	Refs.
Label-SERS	SARS-CoV-2 S protein SARS-CoV S protein MERS-CoV S protein	Anti-spike antibody attached gold nanoparticles	5 min	4 pg/mL	N/A	[104]
	SARS-CoV-2 S protein other influenza viruses	DNA aptamers-Au nanopopcorn	15 min	10 PFU/mL	SARS-CoV-2 lysis solution	[107]
	SARS-CoV-2 S protein	Hollow Au NPs, and magnetic beads	30 min	2.56 fg/mL	SARS-CoV-2 lysis solution	[108]
	Breath volatile organic compounds	Ag nanocubes	5 min	N/A	aerosol	[126]
	SARS-CoV-2 S protein	Au membranes	N/A	17 virus/μL	saliva	[127]
	SARS-CoV-2 S protein HIV-1 P24	AuNPs films	N/A	6.07 fg/mL	untreated saliva	[128]
Label-free SERS	SARS-CoV-2 S protein SARS-CoV S protein	Au nanoneedle array	5 min	80 copies/mL for SARS-CoV-2	Contaminate Water and simulated urine sample	[6]
	SARS-CoV-2 S protein SARS-CoV, MERS-CoV, et al	AuNP array	20 min	N/A	throat swabs or sputum	[109]
	SARS-CoV-2 S protein H1N1 Marburg and Zika virus	field-enhancing metal − insulator antenna	25 min	10^4^ copies/mL	viral lysate solutions	[110]
	SARS-CoV-2 S protein SARS-CoV S protein	SnS_2_	N/A	10^–12^ M for SARS-CoV-2 S protein	PBS	[120]
	SARS-CoV-2 RBD SARS-CoV S RBD	ACE2 mimetic peptide-SERS substrate	≥ 30 min	300 nM for SARS-CoV-2 RBD	PBS	[129]
	SARS-CoV-2 S protein SARS-CoV-2 N protein	Au/Cu nanostar	N/A	8.89 × 10^–9^ M for SARS-CoV-2 S protein	70% ethanol	[121]
	SARS-CoV-2 S protein influenza viruses H1N1	AgNPs/SiNWs	Few minutes	9.3 × 10^−12^ M for SARS-CoV-2 S protein	PBS	[130]
	SARS-CoV-2 Other influenza viruses	DNA aptamers-AgNPs	7 min	5.5 × 10^4^ TCID50/mL for SARS-CoV-2	Mixture of fetal bovine serum, L-Gln, penicillin / streptomycin	[131]

#### Label-SERS for the detection of SARS-CoV-2

**Fig. 6:**
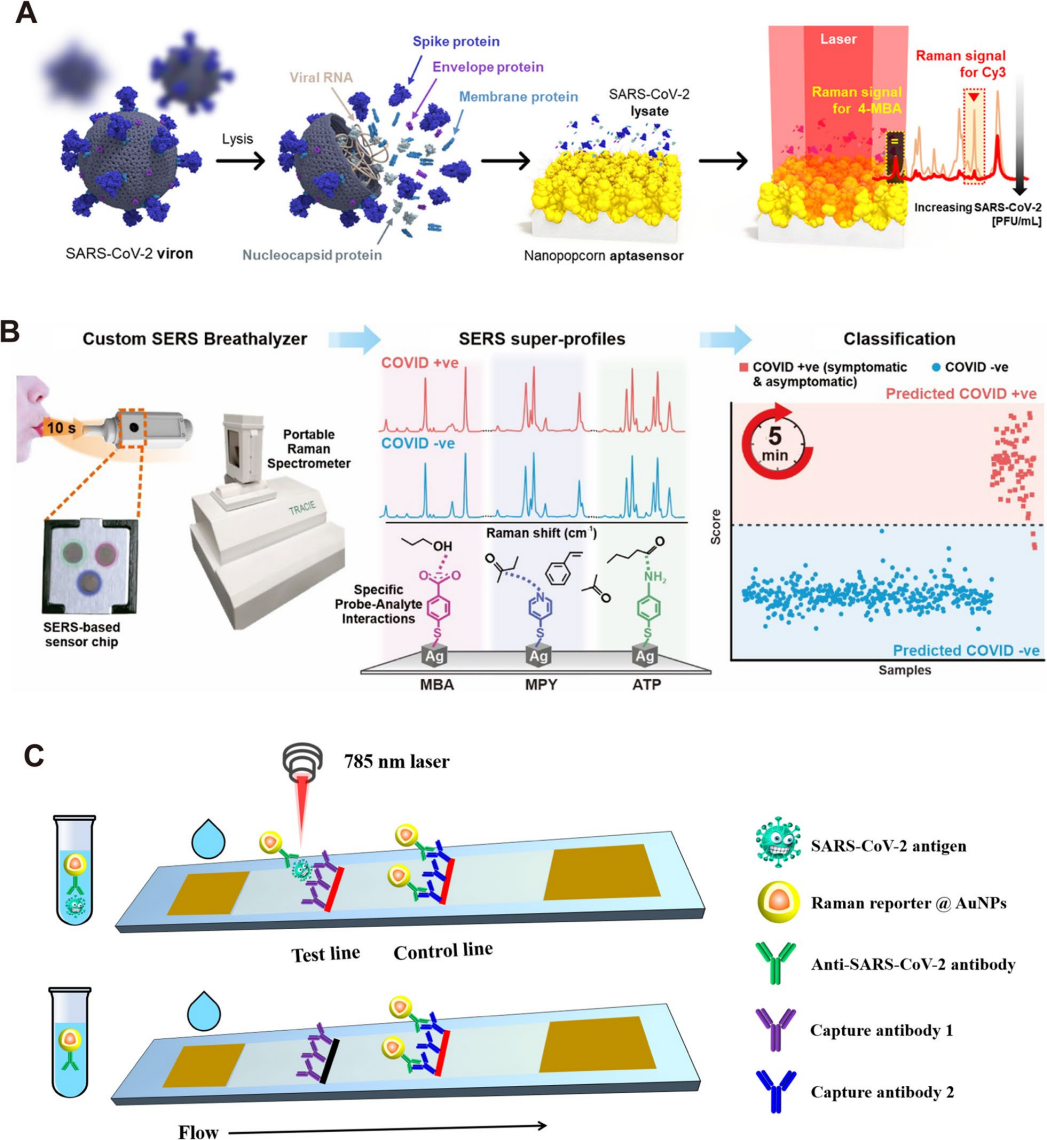
Label-SERS for the detection of SARS-CoV-2. **A** Schematic illustration of the quantitative evaluation of SARS-CoV-2 using the SERS-based aptasensor. After SARS-CoV-2 lysates release the target spike proteins, they are recognized by the aptamer DNAs on the AuNPs surfaces. The spike protein-bound aptamers move away from the AuNPs surfaces, leading to a decreased Raman peak intensity of Cy3 reporters [107]. **B** Experimental procedure for diagnosing the infectiousness of SARS-CoV-2 [126]. **C** Simple illustration of SERS-LFA platform for detecting SARS-CoV-2 antigen. Liquid move via capillary flow on the nitrocellulose membrane. When SARS-CoV-2 antigens are present, they bind to the labeled AuNPs and continue to move until they are captured by the immobilized antibody 1. The labeled control antibodies comigrate until they are captured at the control band. **A** reprinted with permission from Ref. 107, © 2021, American Chemical Society. Figure **B** reprinted with permission from Ref. 126, © 2021, American Chemical Society

The labeling method is mainly used to detect SARS-CoV-2 indirectly by detecting the signal of the Raman-reporter molecule, so the selection of the reporter molecule and the construction of the SERS active substrate are very critical. Nanoscale Au, Ag or composites with strong electromagnetic field enhancement are often used as substrates. 4-mercaptobenzoic acid (4-MBA), 4-aminothiophenol, and rhodamine 6G (R6G) with -SH/-NH groups are often applied as reporter molecules. Strong binding caused by electrostatic interaction or Ag/Au–S/N bonding was used to construct SERS labels. Choo et al. at Chung-Ang University, Korea, were rapidly involved in the detection of the virus after the outbreak of the COVID-19 and constructed several SERS immuno-probes for SARS-CoV-2 detection with an optimal sensitivity of 0.22 pfu/mL [107, 108, 132]. As shown in Fig. 6A, Choo et al. constructed a SERS-based aptasensor platform for monitoring the change in the SERS peak intensity caused by the new binding between the aptamer DNA released from the platform surface and the S protein in the SARS-CoV-2. In general, the SERS-based rapid detection of single virus is restricted to the detection of a single batch of samples. Because the laser device can only serve one chip at a time, detection time will be consumed in the process of looking for viruses. Although SERS sensors are difficult to perform high-throughput detection like PCR facing viruses with low viral loads, SERS has significant advantages in POC (point of care) detection in some specific scenarios (such as customs, airport, express transportation, etc.).

Recently, a research group at Nanyang Technological University designed a SERS-based breathalyzer to monitor the changes in human breath volatile organic compounds (BVOC) to detect SARS-CoV-2 (Fig. 6B) [126]. Upon exposure to breath, molecular receptors with various active chemical functionalities on the SERS sensor form complementary receptor − BVOC interactions such as ion − dipole interactions or hydrogen bonding with the diverse range of BVOCs present. These interactions elicit specific spectral variations to accentuate minute differences in BVOC compositions between COVID-positive and COVID-negative individuals. Participants are simply required to blow continuously into the breath chamber for 10 s and can receive their test result within 5 min, since there is no need for any sample pretreatment. This research is a reference for the application of SERS technology into the applied market. Large-scale fabrication of SERS chips is also critical for large-scale POC detection. Johns Hopkins University, a leading authority on SARS-CoV-2 research in the United States, has developed a novel SERS-based SARS-CoV-2 biosensor using large-area nanoimprint lithography [110]. The biosensor incorporates machine learning technology to improve both detection accuracy and detection speed, making it particularly suitable for large-scale population detection.

In promoting the POC application of SERS sensing, labeling SERS and label-free SERS have their own advantages. Regardless of their detection time, the label-free method saves a lot of cumbersome pre-processing operations. In addition, the label-free method uses less reagents, which is more suitable for the production and storage of sensor chips. Nevertheless, the application of label-free method has to face the interference of various impurity signals in biological fluid. On the one hand, appropriate substrate design is required to reduce the sensitivity to background signals. On the other hand, machine learning based on big data is also essential to identify virus signals [109, 110]. In contrast, the labeling SERS approach yields a simpler spectrum and is more likely to be applied in a generalized manner. More importantly, the same kind of high-sensitivity SERS substrate and corresponding detection protocols can be achieved for different viruses by simply changing the corresponding antibody/aptamer. In addition, the combination of labeling SERS with microfluidic chip or lateral flow assay (LFA) is also a strategy with practical value [106]. Fig. 6C gave a schematic diagram of a SERS-LFA platform for SARS-CoV-2 detection, which is same as the principle of currently used SARS-CoV-2 antigen LFA strip. However, SERS-LFA platform has better sensitivity comparing with visual evaluation and fluorescent-LFA [133, 134]. In the case of SERS-LFA platform, the key issue is a critical improvement of its accuracy using appropriate antibodies (antigen kit) or recombinant proteins (antibody kit) associated with SARS-CoV-2. Consequently, SERS-based LFA can be used to overcome the inherent limitations of traditional LFA, which is of great significance to solve insufficient sensitivity of SARS-CoV-2 antigen detection.

### Tumor detection

#### Detection of tumor markers

**Fig. 7:**
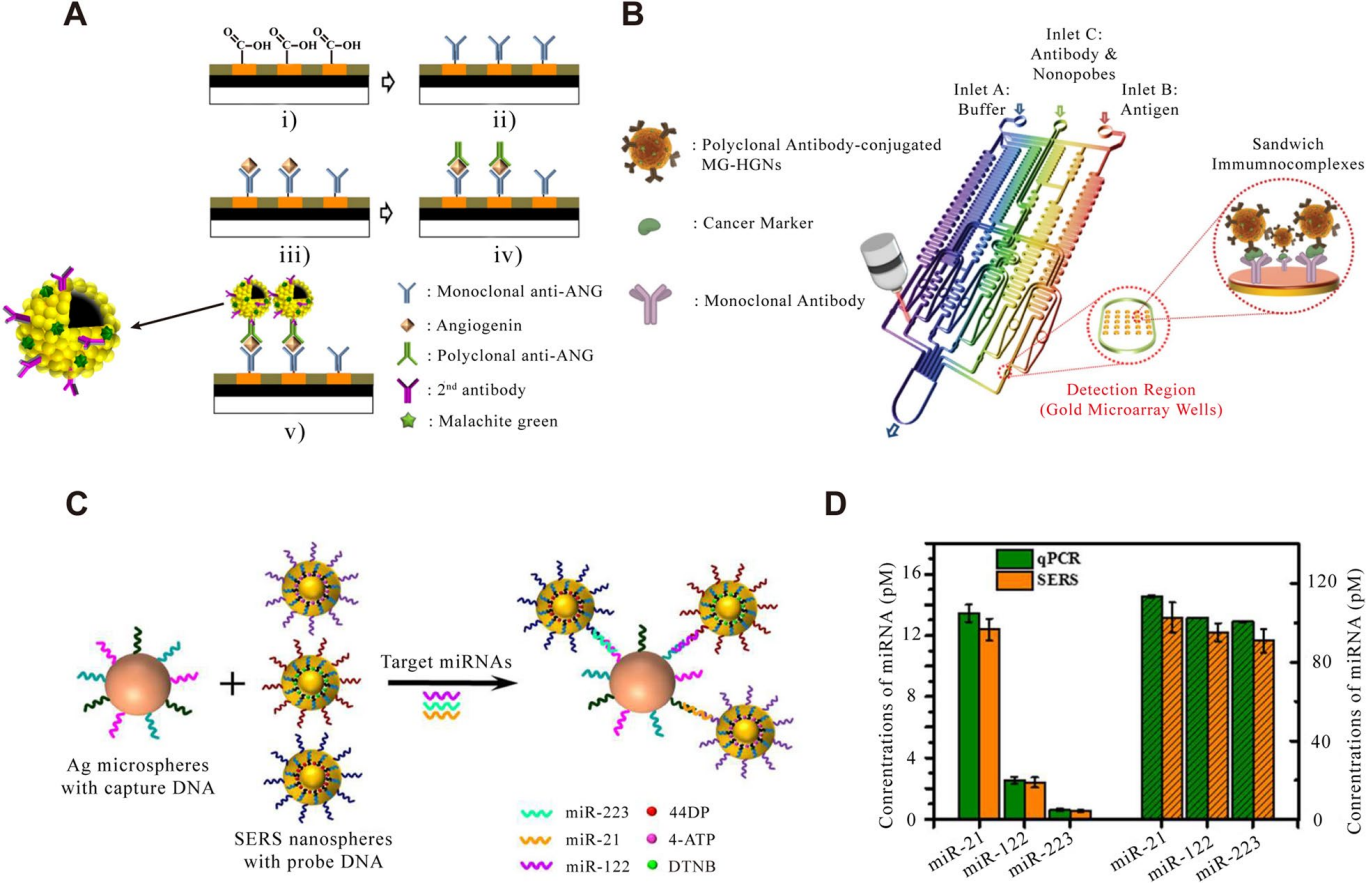
Tumor detection with cancer markers. **A** Schematics of sandwich immunocomplex formation for SERS imaging-based assay: (i) carboxylic acid modification, (ii) antibody immobilization, (iii) capturing of angiogenin antigens, (iv) polyclonal antibody immobilization, and (v) formation of HGN-binding immunocomplexes. [135] **B** Layout of a gold array-embedded gradient chip for the SERS-based immunoassay. The illustrations in the enlarged circles represent the formation of sandwich immunocomplexes on the surface of 5 × 5 round gold wells embedded in the gradient channel. [136] **C** Schematic Illustration of the Multiplex SERS assay for Triple-Target miRNA Detection. **D** Concentrations of miR-21, miR-122 and miR-223 in HepG2 samples measured by the proposed SERS sensor (orange column) and RT-PCR (green column). The left Y-axis represents the concentrations of singlet miRNA detection in cell sample. The right Y-axis represents the concentrations of multiplex miRNA detection in cell sample. Error bars show the standard deviation of three experiments. [143] **A** reprinted with permission from Ref. 135, © 2011, Elsevier. **B** reprinted with permission from Ref. 136, © 2012, Royal Society of Chemistry. **C** and **D** reprinted with permission from Ref. 143, © 2017, American Chemical Society

The application of SERS technology in tumor detection can be divided into in-vivo and in-vitro detection. In-vitro detection is mainly detecting some tumor markers (such as alpha-fetoprotein, carbohydrate antigen, microRNA, carcinoembryonic antigen, and exosomes, etc.) or using some immune complex tags. Alpha-fetoprotein (AFP) is a kind of glycoprotein, which has many important physiological functions, including transport, bidirectional regulation as a growth regulator, immunosuppression, T lymphocyte apoptosis and so on. AFP is closely related to the occurrence and development of liver cancer and many kinds of tumors. It has a high concentration in various tumors and can be used as a positive detection index for various tumors. At present, it is mainly used as a serum marker of primary liver cancer for early screening of tumors [135]. Using SERS technology to detect AFP directly or indirectly from the blood has been widely studied [135–137]. SERS-based detection of cancer markers commonly used labeling methods—using the presence of antigens in body fluids to prepare specific sandwich immune complexes, and then detect Raman reporter on the surface of the complexes. As shown in Fig. 7A, this is a typical case of using labeling-SERS to detect tumor markers in blood [135]. Choo et al. chose AFP and angiopoietin (ANG, a protein associated with angiogenesis of tumor growth) as model protein antigens to detect hepatocellular carcinoma (HCC). The hollow gold nanospheres (HGN) labeled with Malachite Green isothiocyanate (MGITC) were prepared as probes, and the antibodies were immobilized on its surface to target specific antigens. Subsequently, gold-patterned hybrid microarray chips including hydrophilic gold wells and other hydrophobic regions were prepared. Because only the gold patterned area is hydrophilic, while other areas are hydrophobic, the hydrophilic samples are automatically arranged on the surface of gold wells during detecting. Carboxyl groups were then modified in the golden well. The capture antibody was fixed on the surface of the gold well, and the rest of the sites were treated with bovine serum albumin (BSA) to prevent non-specific binding. The antigen is then added and binds to the captured antibody. After washing, the detection antibody was added and bound to the antigen. Finally, HGN with Enzyme-linked secondary antibodies was added and bound to the detected antibody. The detectable dynamic range of SERS imaging (10^–4^-10^–12^ g/mL) is much wider than that of enzyme-linked immunosorbent assay (ELISA) method (10^–6^-10^–9^ g/mL). The labeling method has excellent sensitivity for the detection of tumor markers, but it can be seen from the above steps that such methods usually require tedious procedures. Additionally, all the immune reagents are fixed on the surface of SERS substrates in the air. In general, long-term exposure to air will seriously reduce the biological activity of proteins. Meanwhile, repeated washing to remove non-specific binding proteins makes this immobilization-based determination technique inconvenient. This time-consuming and manually controlled process reduces the attractiveness of the SERS-based gold pattern microarray platform. However, combining SERS technology and microfluidic platform brings great convenience for immunoassay [138]. In another work, Choo et al. combined the above gold pattern microarrays with microfluidic platform to design a programmable fully automatic gradient microfluidic chip. Figure 7B shows a detailed diagram of the gradient microfluidic channel integrated with the embedded gold microarray. Firstly, the carboxylate-terminated self-assembled monolayer was modified on the gold wells to form a hydrophilic surface. The anti-AFP capture antibody was immobilized on the hydrophilic surface of the gold well. Subsequently, the anti-AFP antigen (cancer marker) and anti-AFP polyclonal antibody composite MGITC-HGN (functional nanoprobe) were injected into entrance B and C in turn, flowing downward and used to form sandwich immune complex on gold pattern microarray. The total determination time of the microfluidic chip from continuous dilution, incubation and washing to SERS detection is less than 60 min. Because all immune complex formation and detection can be automatically controlled by well-designed microfluidic channels, this new microfluidic detection technology based on SERS is expected to become a powerful clinical tool for rapid and sensitive detection of cancer markers.

AFP is usually used for auxiliary examination of human tumors in clinic, because there are many factors for the increase of AFP, such as pregnancy, inflammation, etc. Therefore, it is very important to obtain more valuable information from human blood by SERS method. Carcinoembryonic antigen (CEA) is a glycoprotein produced by cancer tissues, which can cause immune response in patients and can be widely found in digestive system cancer of endodermal origin [139, 140]. CEA has been used as a specific marker for early diagnosis of colon and rectal cancer in the past. After a large number of clinical practices, it was found that CEA increased not only in gastrointestinal malignant tumors, but also in breast cancer, lung cancer and other malignant tumors. Although CEA cannot be used as a specific index for the diagnosis of some malignant tumors, it still is a broad-spectrum tumor marker that has important clinical value in differential diagnosis, disease monitoring and curative effect evaluation of malignant tumors. Choo et al. achieved an LOD of 1–10 pg/mL for CEA using magnetic beads, in combination with the developed HNG immune complex [141]. This value is about 100–1000 times more sensitive than that of ELISA, and the whole testing process can be completed within an hour.

MicroRNAs (miRNAs) are a class of endogenous small RNA with a length of about 20–24 nucleotides. The combination of several miRNAs can also tightly regulate the expression of a certain gene. MiRNAs show different expression levels in cancer, which can affect cell transformation, carcinogenesis, and metastasis. Abnormal expression of miRNA was found in all types of tumors, including pancreatic cancer, lung cancer, prostate cancer, colorectal cancer, triple negative breast cancer and osteosarcoma [142]. The ultra-high sensitive detection of tumor-specific circulating miRNAs is of great significance for early diagnosis and monitoring of cancer. Zhou et al. proposed a sandwich hybridization assay based on multiple SERS for the detection of specific miRNAs, miRNA-21, miRNA-122 and miRNA-223 in HCC [143]. As shown in Fig. 7C, the ability of SERS assay to detect multiple miRNA targets was studied using a general multiple analysis strategy. Three types of SERS reporters (three non-fluorescent Raman reporter molecules DTNB, 4-ATP and 44DP labels) were functionalized with corresponding probe DNA (complementary sequence with specific target miRNA) to prepare SERS nanoprobes. Moreover, the capture DNA of three miRNA targets was co-assembled on AgNPS to form multiple capture substrates. Subsequently, the mixture of the target miRNAs was captured by multiple substrates and hybridized with the corresponding SERS nanoprobes to form a multi-sandwich hybrid complex for analyzing. The LOD of this scheme for a specific miRNA is as low as 10 fM. In particular, this method provides a way to detect three kinds of miRNAs simultaneously in a single SERS experiment with high sensitivity and specificity. As shown in Fig. 7D, this method is further applied to detect single or multiple target miRNAs in actual human HCC samples (HepG2) and the results are compared with quantitative real-time polymerase chain reaction (RT-PCR). The results obtained by SERS sensor are in acceptable agreement with those obtained by RT-PCR, which indicates that SERS sensor can be used for sensitive detection of multiple miRNAs in cells. In SERS applications, Fe_3_O_4_ magnetic nanoparticles are a common part of functional nanoparticles to separate and enrich target biomarkers. Pang et al. designed a functional Fe_3_O_4_@Ag magnetic nanoparticle biosensor for capturing and ultra-sensitive detecting miRNAs in the total RNA extract of cancer cells [144]. Through endonuclease duplex specific nuclease (DSN) selectively cleaving DNA probes of DNA/miRNA double strand, a target miRNA molecule can rehybridize thousands of DNA probes to trigger the signal amplification cycle. The LOD of this sensor is 0.3 fM, which is nearly three orders of magnitude lower than the traditional fluorescence-based DSN biosensor (~ 100 fM). Notably, combined with the labeling method, the scheme can be used to detect miRNAs in exosomes and supernatant plasma for pancreatic cancer diagnosis, and the detection limit is 1 aM with single base recognition ability [145]. In fact, with the continuous development of new materials, in addition to traditional noble metal SERS sensors, some semiconductor-based SERS substrates can also achieve miRNA detection with similar sensitivity [142].

**Fig. 8:**
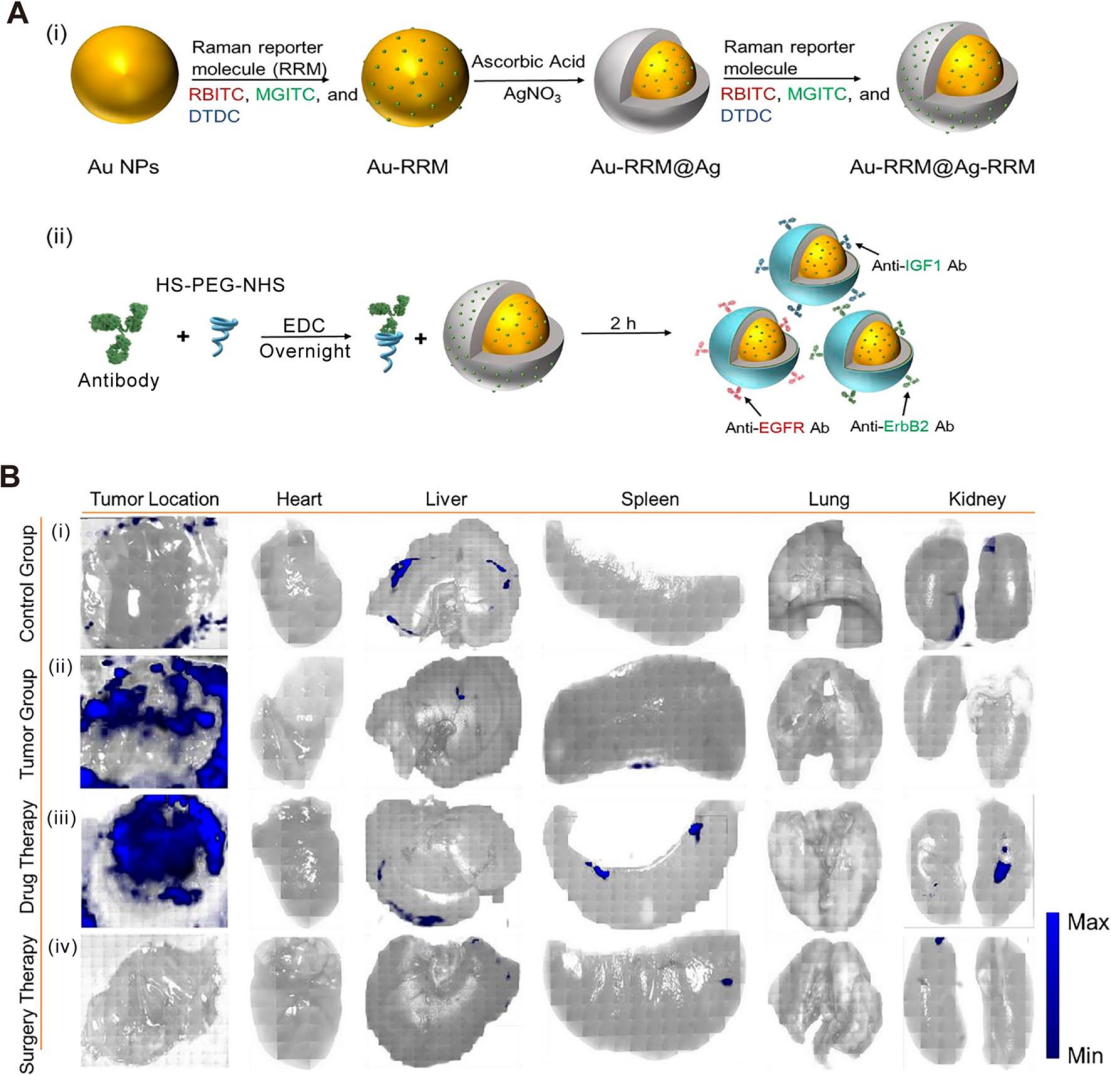
Cancer cell targeting and spectroscopic detection by using antibody-conjugated SERS nanoparticles. **A** Schematic illustrations (i) for the fabrication of three different Raman reporter-adsorbed Au–Ag core–shell nanoparticles and the conjugation of PEGylated antibodies on the surface of the above Au–Ag core–shell nanoparticles. [148] **B** The evaluation of before and after treatment towards the tumors based on SERS imaging. [148] (i) Control group: SERS imaging of right axilla of healthy nude mice and organs. (ii) Tumor group: SERS imaging of breast tumor and organs without any treatment. (iii) Drug therapy group: SERS imaging of breast tumor and organs after tamoxifen treatment for 15 days. (iv) Surgery therapy group: SERS imaging of breast tumor and organs after surgery. Figure **A** and Figure (**B** reprinted with permission from Ref. 148, © 2023, Elsevier

Some research suggests that there is high or abnormal expression of epidermal growth factor receptor (EGFR) in many solid tumors. EGFR is related to tumor cell proliferation, angiogenesis, tumor invasion, metastasis, and inhibition of apoptosis [146, 147]. Research based on EGFR biomarkers will be a breakthrough in the detection and treatment of early cancer. However, the weak adsorptivity and cellular aversions to templates led to inadequate capability of traditional SERS template to discern the biomarker. Venkatakrishnan et al. optimized the LOD of TiO_2_ quantum probe to 1 nM by introducing oxygen vacancy into TiO_2_ [146]. Using this quantum probe, EGFR peptides and higher lipid content than fibrous cells can be identified in breast cells. It is generally considered that the labeling method is not suitable for direct in-situ detection in-vivo. When nanoparticles are used in biomedical applications, some surface functionalization or protective coating is usually required for SERS probes, which we discussed in the previous chapter. As shown in Fig. 8A, a typical design of a SERS probe for in-vivo tumor detection is given here [148]. The SERS probes are made of Au@Ag core–shell nanoparticles, decorated with Raman reporter double-layer on the surface of Au core and Ag shell with functional polyethylene glycol (HS-PEG-NHS) layer for the antibody conjugation. PEG is a non-toxic hydrophilic polymer, which is usually used to improve the biocompatibility of nanoparticles [149]. The design of a double-layer Raman reporters creates significantly enhanced Raman signals for ultrahigh sensitivity. After equipping the specific antibodies for growth factor reporters, the SERS probes can actively target the tumor cells for precise detection of phenotypic biomarkers and therapeutic evaluation. As shown in Fig. 8B, after intravenous injection with SERS probes for 12 h, the in-vivo SERS imaging of tumor location was obtained. Then, the mouse was sacrificed and major organs were excised and imaged in vitro, whereas only the liver and kidney displayed relatively high SERS signals. These results might be contributed to the metabolism of SERS probes in the liver and spleen. In contrast, the xenotransplanted tumor showed very high SERS signal intensities while major organs remained low signal intensities, indicating the active-targeting capability of SERS probes (Fig. 8B, ii). After anticancer drug tamoxifen treatment for 15 days, the SERS signal-positive areas decreased compared to the tumor group, demonstrating its inhibition effect towards the breast tumor (Fig. 8B, iii). As shown in Fig. 8B, iv, mouse treated with standard surgery displayed almost no significant SERS signals, indicating the complete tumor elimination. The above results show that SESR technology for targeted diagnosis of tumors in vivo can provide a full process of monitoring from detection, treatment to prognosis. In addition, SERS probes will not have harmful effects on normal tissues. Nie et al. showed a polyethylene glycol (PEG) functionalized gold nanoparticles for targeting and detecting EGFR positive tumors [150]. SH-PEG-COOH binds covalently with ScFv antibody (a ligand that binds EGFR with high specificity and affinity). The SERS spectra obtained by incubating ScFv-conjugated gold nanoparticles with human cancer cells. As a result, human head and neck cancer cells (Tu686) were EGFR positive (10^4^–10^5^ receptors per cell), and showed strong SERS signal. Moreover, the targeted nanoparticles have no biotoxicity or other complications. When SERS gold nanoparticles were injected through the tail vein of mice, the nanoparticles were targeted to bind to EGFR positive tumor cells and located in intracellular organelles, such as endosomes and lysosomes, while accumulation was hardly observed in the brain, muscle, or other major organs. Therefore, due to the high sensitivity and safety of SESR technology, it is of great value in the early detection of tumors or monitoring of treatment.

Finally, it is necessary to mention the exosome-based liquid biopsy method developed in recent years**.** Exosomes, together with circulating tumor cells (CTCs) and circulating tumor DNA (ctDNA), are the three main biomarkers of fluid biopsy, which can provide a non-invasive solution for early detection, diagnosis, and prognosis of cancer patients. Exosomes are derived from microvesicles formed by invagination of lysosomal particles and widely exist in biological body fluids. They carry chemical information that reflects cellular characteristics, including nucleic acids, proteins, lipids, amino acids and metabolites [151, 152]. The collected Raman signal of exosomes has poor homogeneity and reproducibility on bare SERS substrate duo to the large size of exosomes (100–200 nm) [36]. Due to the large volume and complex surface composition of exosomes, the attachment direction of exosomes will be different when combined with SERS substrate, resulting in different enhanced positions. On the one hand, it is interfered by impurity signals in body fluids. Hence, the following two strategies are usually adopted in tumor detection based on exosomes. The first is the labeling method. It detects the Raman reporter molecule labeled on SERS tags to indirectly detect targets. The advantages of the labeling method are clear signal, low LOD, and sensitivity up to 500 particles/mL [153]. Moreover, the sensitivity of the biosensor can be adjusted by modular SERS labeling design, facilitating on-demand design and optimization of the biosensor for differentiated applications [154]. However, the operation of the labeling method is tedious and prone to misjudgment. Secondly, the exosomes can be captured and fixed by modifying the surface of SERS substrate. Some proteins on exosomes surface can be used as anchoring sites, such as members of the tetraspanins family (CD37, CD53, CD63, CD81 and CD82), MIF, GPC1, EGFR, Lamp-2b, Glycosylphosphatidylinositol (GPI), etc. The method of capturing exosomes from serum combined with microfluidic technology can achieve higher detection efficiency. In addition, SERS probes can also be designed directly to hybridize with nucleic acids in exosomes to detect tumor information [155]. As shown in Table 3, we compared some latest studies using different methods or different SERS substrates for exosomes detection. In general, the SERS enhancement of semiconductors mainly comes from charge transfer with high selectivity. Therefore, the signal uniformity of semiconductor templates is obviously better than that of noble metal. We found that the semiconductor-based SERS substrate has good stability and specificity even for macromolecules, such as exosomes [156, 157]. The rapid development of semiconductor materials may point out a new idea for biomarker detection, the key of which is to develop the semiconductor SERS templates with high sensitivity.

**Table 3:** Comparison of different methods for exosome recognition

Type	Substrate	Detection limit	Methods	Refs.
Colorimetric biosensors	Paper-based lateral flow biosensor	8.5 × 10^5^ particles/μL	Antibody enrichment	[158]
Fluorescent biosensors	Solution-based biosensor	4.8 × 10^4^ particles/μL	Magnetic enrichment	[159]
SPR biosensors	Gold chip-based biosensor	8.28 × 10^3^ exosomes/μL	Antibody microarrays	[160]
Electrochemical biosensors	Electrode-based biosensor (carbon electrode)	100 particles/μL	Magnetic enrichment	[161]
SERS sensor	AuNR array biosensor	5.3 × 10^3^ particles/μL	Labeling Method	[162]
SERS sensor	Au nanostar biosensor	27 particles/μL	Labeling Method	[163]
SERS sensor	Ag@Au Nanoparticles	1 particles/2μL	Labeling Method	[153]
SERS sensor	MoS_2_-AuNSs aptamers	17 particles/μL	Labeling Method	[79]
SERS sensor	Au nanostar	2.4 particles/μL	Labeling Method	[154]
SERS sonsor	Au nanoparticles	10^6^ particles/μL	Label-free Method	[36]
SERS sonsor	Ag/BP-NS biosensor	5 × 10^4^ particles/μL	Label-free Method	[157]
SERS sonsor	Nano-porous gold	100 particles/μL	Label-free Method	[164]

#### In-situ detection of tumors

Gene/DNA is the “architectural drawing” of the human body, which carries all the genetic information of the human body. When some genes are abnormal, it will affect all kinds of physiological activities of the human body, and in serious cases it will lead to disease. Gene mutation is the root cause of cancer. In order to obtain accurate genomic information from the origin of cancer, detection methods with non-invasive, unmarked, high sensitivity and ability to capture multiple gene information are needed. The application and development of new nanostructured materials play an important role in the application of SERS. Compared with noble metals, semiconductor-based SERS materials have the advantages of biocompatibility, low cost, chemical stability and high adsorption, so they are more suitable for in-situ detection as SERS substrate. The biggest obstacle faced by semiconductor SERS substrates is the lack of sensitivity. As more and more new semiconductor materials or structures are discovered as research progresses, the sensitivity of semiconductor substrates continues to improve. Some of the semiconductor substrate materials that have been studied are listed in Table 4. It can be seen that the detection limits of some materials have reached femtomolar level with enhancement factors of 10.^10^. Venkatakrishnan and Tan et al. have fabricated various quantum size semiconductor probes (including ZnO, Si@SiO_2_, TiO_2_ and organic semiconductors) by femtosecond laser ablation and have done a lot of work in in-situ diagnosis of cancer cells in-vivo/in-vitro [10, 21, 146, 165–167].

**Table 4:** Performance of some high sensitivity semiconductor-based SERS substrates

Materials	Probe molecule	LOD	EF	Refs.
GaP	Copper Phthalocyanine	N/A	7 × 10^2^	[51]
WSe_2_	Copper Phthalocyanine	N/A	10^2^	[168]
GaN	Basic Fuchsin	N/A	1 × 10^7^	[169]
CdTe	4-Mercaptopyridine	1.8 × 10^–3^ M	1 × 10^4^	[52]
InAs	Simulation calculation	N/A	10^10^–10^11^ (THz excitation)	[170]
CdSe	4-Mercaptopyridine	10^–1^ M	N/A	[171]
CdSe-TiO_2_ IOS Film	Methylene Blue	7 × 10^−9^ M	1.46 × 10^5^	[172]
ZnO/Ag@Au	Rhodamine 6G	10^−10^ M	1.48 × 10^9^	[173]
ZnS	4-Mercaptopyridine	9 × 10^−3^ M	10^3^	[174]
ZnSe	4-Mercaptopyridine	10^−3^ M	2 × 10^6^	[175]
Nb_2_O_5_	Methyl Violet	10^−8^ M	2.09 × 10^7^	[176]
Ta_2_O_5_	Methyl Violet	9 × 10^−9^ M	2.2 × 10^7^	[177]
TiO_2_	Rhodamine 6G	1 × 10^−7^ M	1.20 × 10^6^	[24]
CdS	Benzenethiol	N/A	N/A	[178]
CuO	Rhodamine 6G	1 × 10^−8^ M	N/A	[179]
AgFeO_2_	Rhodamine 6G	1 × 10^−7^ M	5.1 × 10^5^	[180]
ReS_2_	Copper Phthalocyanine	N/A	10	[181]
W_18_O_49_	Rhodamine B	1 × 10^−7^ M	N/A	[182]
V_2_O_5_	Rhodamine 6G	1 × 10^−8^ M	N/A	[183]
CuTe	Nile Red	N/A	10^6^	[184]
Si (H-SiNWs)	Rhodamine 6G	1 × 10^−6^ M	10^2^	[185]
Ge (H-GeNT)	Rhodamine 6G	1 × 10^−6^ M	10^2^	[185]
Cu_2_O	4-Mercaptobenzoic	10^–3^ M	5.36 × 10^5^	[186]
Pb_3_O_4_	4-Mercaptopyridine	10^–7^ M	N/A	[187]
Black Phosphorus	Crystal Violet	10^–5^ M	2.14 × 10^5^	[188]
Graphene	Rhodamine B	∼10^–8^ M	10^3^	[189]
MoS_2_	4-Mercaptopyridine	10^–3^ M	3 × 10^5^	[190]
TaSe_2_	Rhodamine 6G	10^–10^ M	1.5 × 10^5^	[191]
Ti_3_C_2_	Rhodamine 6G	10^–11^ M	3.82 × 10^8^	[192]
SnS_2_	Methylene Blue	10^–13^ M	3.0 × 10^8^	[120]
MoTe_2_	Rhodamine 6G	4 × 10^–14^ M	6.2 × 10^9^	[193]
Ti_2_N	Rhodamine 6G	10^–15^ M	10^12^	[194]
Re-WSe_2_	Rhodamine 6G	5 × 10^–15^ M	2.0 × 10^9^	[195]
WTe_2_	Rhodamine 6G	4 × 10^–15^ M	4.4 × 10^10^	[193]
Ag/BP	Rhodamine 6G	10^–20^ M	1.01 × 10^11^	[157]

**Fig. 9:**
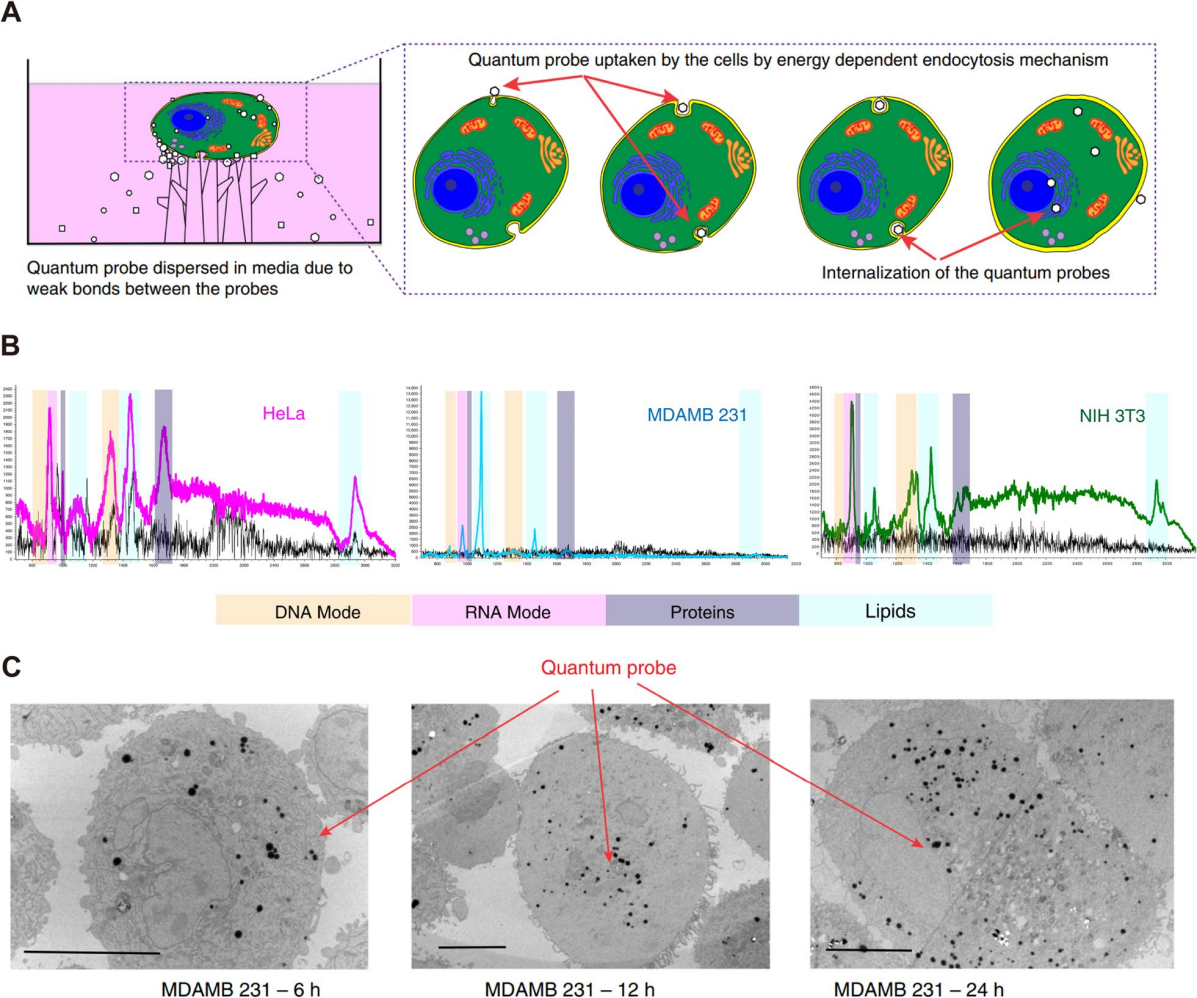
Cellular uptake mechanism of the quantum probe. **A** Schematic representation of the endocytosis mechanism. **B** Enhanced SERS signal for cancer and non-cancer cells. Magenta, cyan and green represent SERS signal and black spectra for non-SERS response. **C** Cell TEM reveal time-dependent cellular uptake of the quantum probes. Scale bar $=10 \mu m$. [196] Figure (**A**–**C**) reprinted with permission from Ref. 196, © 2018, Springer Nature

Tan et al. observed that once the size of the semiconductor probe is reduced to the quantum scale, the SERS enhancement increases exponentially and is easily ingested by cells through endocytosis [196]. As shown in Fig. 9A, ZnO quantum probes were prepared on nano-dendritic platform by femtosecond pulsed laser ablation. The cells can adhere to the 3D nano-dendritic platform, and the quantum probe is then ingested by the cells through the endosomes. As shown in Fig. 9B, the quantum probe can obtain enhanced Raman signals from three types of cells, including breast cancer (MDAMB231), cervical cancer (HeLa) and non-cancerous (NIH3T3) cells. Meanwhile, ZnO quantum probe can simultaneously obtain DNA, RNA, protein and lipid signals. This is due to the fact that many quantum probes are dispersed in lysosomes throughout the cytoplasm, which can be seen in the cell membrane and the whole cytoplasm. As shown in Fig. 9C, with the extension of incubation time, the quantum probe is gradually dispersed in the whole cell. According to Raman spectra analysis (Fig. 9B), the main difference between cancer cells and non-cancer cells is the overexpression of cholesterol and cholesterol esters, which are characteristic lesions of cancer caused by mitochondrial membranes. In addition, the cancer cells show a more obvious bending vibration pattern of CH_2_ (1450 cm^−1^) in malignant tissues. The sensitivity of this method can also reach the level of single cell.

Although SERS offers the enough ultra-sensitive and multiplex detection attributes to get a holistic picture of epigenetic landscape, the interaction of existing SERS probes with DNA greatly alters the native structure of DNA leading to inappropriate diagnosis [167]. In particular, positively charged SERS probes show high toxicity, which can easily lead to DNA damage, apoptosis and cell death. This is due to the fact that the nucleus carries a large number of nucleic acids and proteins, which can effectively bind positively charged nanoparticles [197]. Afterwards, Venkatakrishnan and Tan et al. used the designed quantum organic semiconductor (QOS) to analyze genomic DNA isolated from four different cell lines, including fibroblasts (NIH3T3), breast cancer (MDA-MB231), pancreatic cancer (AsPc-1) and lung cancer (H69-AR), and verified the practicability of untagged QOS [167]. The base composition of DNA and methylation markers can be collected in a single test. The molecular differences of genomic DNA between cancer cells and non-cancer cells were determined by multivariate statistical analysis. Based on the detection of cancer cells using Raman spectroscopy, we usually expect to achieve accurate molecular level analysis in-vitro, which is helpful for the early diagnosis and prognosis of cancer. The Si@SiO_2_ quantum probe prepared by Tan et al. can also be endocytosis to realize the in-situ detection of Hela cells [165]. Using PCA-LDA to compare the Raman spectra of cancer cells and normal mammalian cells (fibroblasts) confirmed the significant differences in amide (III, V), phenylalanine and tyrosine composition. Compared with healthy and dead HeLa cells, a lack or sharp drop in phenylalanine or tyrosine concentrations was found to be a sign of cell death. The results show that it is possible to detect early HeLa cancer based on molecular information rather than morphological characteristics. Using SERS diagnosis can detect the pathological changes before the morphological changes as early as possible, so as to obtain better treatment results. As mentioned above, fluid biopsy is also a non-invasive analysis, usually using exosomes, CTCs or ctDNA in body fluid to detect mutated genes in cancer [198]. BRAF^V600E^ mutation is one of the most valuable tumor markers in fluid biopsy. Crucially, BRAF^V600E^ is also an actionable mutation which could be arrested by clinically beneficial drug inhibitors. Dey et al. achieved circulating BRAF^V600E^ detection at the molecular level of DNA and protein in simulated melanoma plasma samples in the form of liquid biopsies [199]. In addition to the detection of primary tumors, Venkatakrishnan and Tan also reported the detection of metastatic cancer cells for the first time using the prepared semiconductor quantum probes [166]. This probe may also be used for CTCs detection in the near future.

#### SERS imaging for tumor cell

Although SERS technology has traditionally been used as a tool for in-vitro analysis, SERS imaging has shown great potential in the field of medical imaging in the past decade. Compared with other imaging methods, SERS imaging has some outstanding advantages. Medical imaging of SERS nanoprobe can not only produce higher sensitivity and signal specificity, but also provide a variety of functions at the same time, such as SERS imaging, drug transport, real-time monitoring of prognosis, etc [200–202]. The selection of nanoprobes in SERS imaging is very important: (1) the selected material must have high SERS enhancement; (2) the components of nanoparticles should have adequate biocompatibility to avoid the potentially toxic elements or surfactants as much as possible; (3) nanoparticles are usually encapsulated to maintain the unique Raman fingerprint and detection sensitivity [203]. Consequently, gold nanomaterials are most frequently used SERS probes because of their outstanding surface plasmon resonance effect, structural tunability, low acute toxicity and good biocompatibility. Other SERS probes also include some semiconductor nanomaterials, two-dimensional nanosheets, fluorescent quantum dots, carbon nanomaterials, magnetic nanoparticles, etc [204]. SERS imaging can also be divided into label-free and labeling methods. Considering the weak Raman intensity of some biomolecules and the complex biological environment, labeling method is more often used in SERS imaging research [200].

**Fig. 10:**
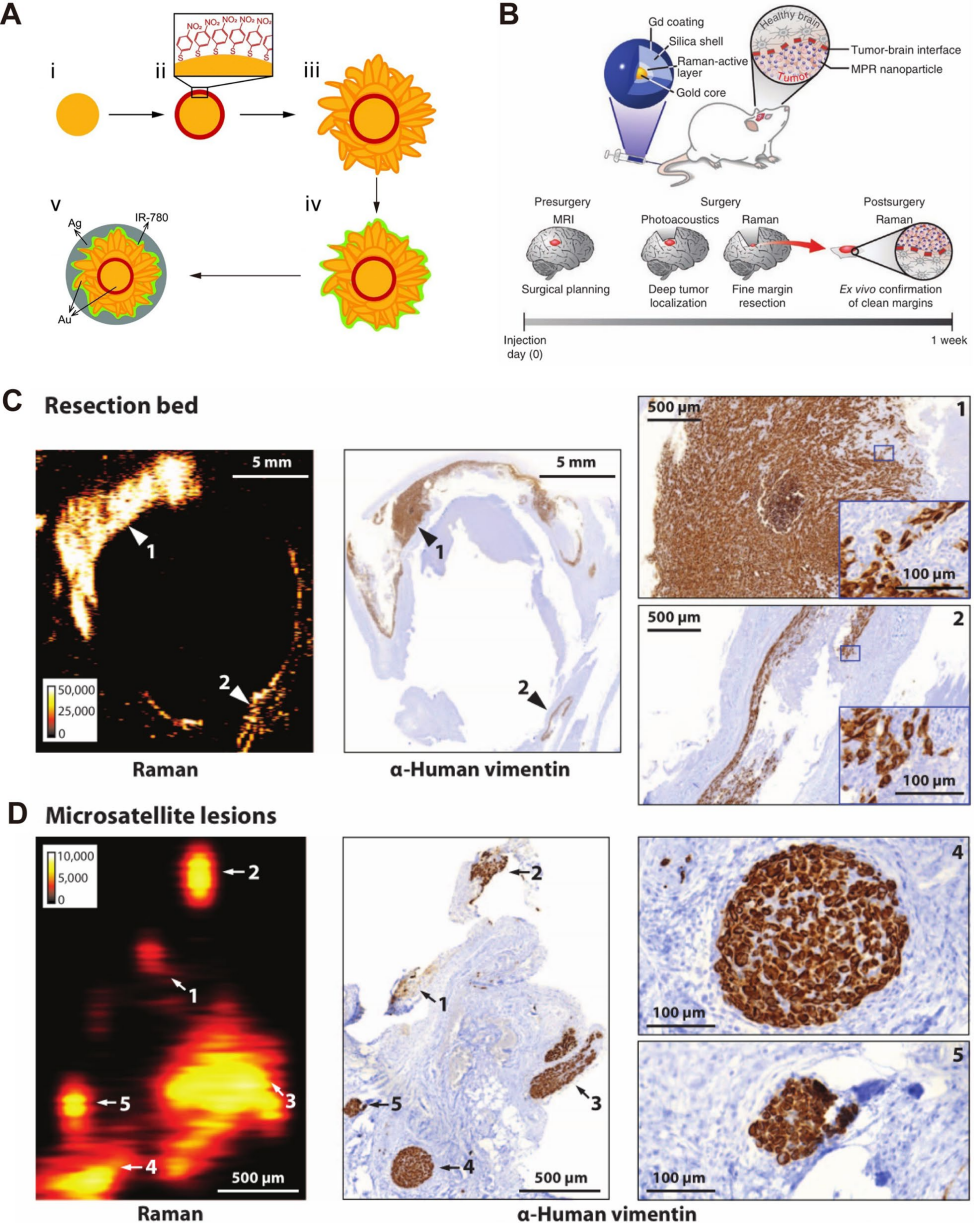
Imaging of cancer with microscopic precision using SERS nanoparticles. **A** Schematic synthesis process of GERTs, including (i) Au cores, (ii) 4-nitrobenzenethiol (4-NBT) modified Au cores, (iii) gap-enhanced Raman tags with a petal-like shell (P-GERTs), (iv) IR-780 modified P-GERTs and (v) GERTs. [207] **B** MPRs are injected intravenously into a mouse bearing an orthotopic brain tumor. As the nanoparticles circulate in the bloodstream, they diffuse through the disrupted blood–brain barrier and are then sequestered and retained by the tumor. The MPRs are too large to cross the intact blood–brain barrier and, therefore, cannot accumulate in healthy brain. [215] **C** SERS image of resection bed was acquired after surgical excision of tumor bulk (left). Resection was guided by white light only, with surgeon blinded to SERS images. Immunohistochemistry staining for human vimentin confirmed that SERS-positive signal (arrows 1 and 2) represented microscopic residual cancer at margins of resection bed (middle). Immunohistochemistry images on right are magnified views of areas indicated with arrows 1 and 2. **D** SERS image of locoregional tumor micrometastases. The multiple small foci of Raman signal (arrows 1 to 5) were found approximately 10 mm away from the margins of the bulk tumor. As confirmed by immunohistochemistry (middle), each of these 5 foci correlated with a separate tumor cluster (vimentin +) as small as 100 $\mu m$ (micrometastases). Images on far right are magnified views of the metastases labeled 4 and 5. [216] **A** reprinted with permission from Ref. 207, © 2020, Royal Society of Chemistry. **B** reprinted with permission from Ref. 215, © 2012, Springer Nature. **C**, **D** reprinted with permission from Ref. 216, © 2017, American Chemical Society

The signal intensity of the SERS probe has an important impact on the quality of SERS imaging. Conventional spherical nanoparticles will lead to reduced Raman signals at lower probe concentrations due to the reduced number of hot spots between the particles. Therefore, researchers usually use nanotags with core–shell structures or "gold nanostars" with synaptic structures for SERS bioimaging [205]. Professor Jian Ye's team from Shanghai Jiaotong University has made many outstanding achievements in SERS probe design, tumor marker identification, biological tissue imaging and machine/depth-based learning for bio-spectral identification [205–210]. As shown in Fig. 10A, Ye et al. designed an ultrabright gap-enhanced Raman tags (GERTs) with strong electromagnetic hot spots from the interior sub-nanogap and external petal-like shell structures, larger immobilization surface area, and Raman cross sections of reporter molecules [206, 207]. These GERTs reach a Raman enhancement factor beyond 5 × 10^9^ and a detection sensitivity down to a single-nanoparticle level. It is embedded with IR-780 NIR resonance reporter and can be used for long-term and high-speed live cell tracking imaging due to reduced photodamage to cells. The combination of this SERS probe with transmission Raman spectroscopy (TRS) also enables the non-invasive and light-safe detection of "phantom" lesions hidden deep in biological tissues [208]. Due to the strong optical scattering and absorption in biological tissues, Raman signal is usually limited by the shallow depth of tissue penetration, which largely limits its application in in-vivo biomedical detection of deep lesions. The combination of GERTs and TRS enables non-invasive detection with high tissue penetration capability. The protocol achieved a 14-cm-thick tissue penetration as well as in-vivo imaging of tumors in an unshaven mouse at a clinically safe laser density. However, biocompatibility, biosafety, and targeted tumor accumulation of GERTs are also important in biomedical applications.

In order to improve the biocompatibility of SERS probes, a SiO_2_ or polymer protective layer is usually coated on the surface of the labeled nanoparticles [201, 211]. One of the most common surface modification molecules is PEG, which can not only prevent excessive aggregation caused by surface charge of nanoparticles, but also confer increased stability within a variety of microenvironments [202]. A typical route for nanoparticle modification was demonstrated by Nie et al [212]. Nanoparticles with a certain size were synthesized and modified with Raman reporter molecules. The nanoparticles were then coated with a SiO_2_ or PEG shell, and the surface can be further functionalized with a targeting agent. There are two general routes to achieve deep tissue imaging in animals: passive accumulation and active targeting [213]. For example, as mentioned earlier, Nie et al. targeting EGFR through monoclonal antibody fragment-modified gold nanoparticles is an active targeting imaging approach [150]. However, many studies have shown that the nanoparticles do not require specific targeting moieties to perform robust tumor imaging [214–216]. In fact, nanoparticles within a certain size range tend to accumulate specifically in cancer tissue but not in normal tissues. This accumulation of nanoparticles without a specific targeting moiety is generally attributed to the so-called enhanced permeability and retention (EPR) effect [217–219]. Among the various requirements for and factors influencing the EPR effect, the most important is having a molecular size larger than 40 kDa [220]. The EPR effect results in less delivery of macromolecular drugs to normal tissues, so the systemic toxicity is less. Because all of these nanoprobes exhibit the EPR effect, these developments are very beneficial in the more effective treatment and sensitive diagnosis of tumors and inflamed tissues. Kircher’s team developed a unique triple-modality magnetic resonance imaging–photoacoustic imaging–Raman imaging nanoparticle (MPR nanoparticle) can accurately delineate the margins of brain tumors in living mice both preoperatively and intraoperatively [215]. The MPR nanoparticle is a 60 nm gold core surrounded by a thin Raman-active layer that is protected by a 30 nm silica coating. The silica coating was further functionalized with maleimide-DOTA-Gd. An ideal SERS probe would be sequestered and retained by a tumor for a long enough period that a single injection of the agent would facilitate both preoperative and intraoperative imaging. As shown in Fig. 10B, the MPR probe can be accurately retained in the tumor tissue with only once injection, and the probe can be detected in the tumor during the operation a few days later. This strategy enables (1) whole brain tumor localization for preoperative and intraoperative macroscopic delineation, (2) high spatial resolution and three-dimensional imaging using photoacoustic imaging, and (3) high sensitivity, high specificity and high-resolution surface imaging of tumor edges using Raman imaging. Interestingly, SERS imaging is able to detect residual tiny cancer foci in resection beds that is not detectable with the unaided eye, which puts forward a high requirement for the LOD of SERS probe. Then Kircher et al. designed a gold nanostar SERS probe with extremely high sensitivity with a LOD of 1.5 fM. As shown in Fig. 10C, D, the microscopic foci at the margins of the resected bulk tumor (Fig. 10C) and locoregional micrometastases (Fig. 10D) were detected by the gold nanostar SERS probes, respectively. In both cases, the surgeon was unable to detect residual tumor using conventional methods (white light illumination). This ultrasensitive SESR probe is able to image multiple tumor types, including breast cancer, prostate cancer, pancreatic cancer, and different types of sarcoma. The specific targeting epitopes of different tumors in clinical diagnosis may not be identified in the early stage of diagnosis. In contrast, this EPR-based macromolecular tumor therapy, which does not use targeting ligands, has broader advantages over tumor-targeting antibodies and can be applied to a broader range of tumors.

### Pesticide detection

Pesticides are indispensable in modern agricultural operations. Excessive use of pesticides leads to pesticide residues in agricultural by-products, which not only pollutes the environment but also poses a serious threat to human health. Based on their chemical structures and functionality, synthetic pesticides are classified into five classes: organochlorine, organophosphate, carbamate, neonicotinoid and pyrethroid [221]. The detection methods of pesticide residues require high sensitivity and repeatability, among which the most widely used method is standard chromatography. At present, some methods have been proposed for detecting trace pesticide residues, including electrochemical detection, capillary electrophoresis, and immunoassay. However, these techniques typically require time-consuming sample extraction, purification or pre-concentration before analysis, and are especially not suitable for on-site detection. Compared with these methods, SERS has the following advantages: (1) it is suitable for various forms of sample testing, which can be used for in-situ sampling detection; (2) the detection time is fast and the whole detection, analysis and judgment process can be completed within 10 s; (3) the sensitivity is high and LOD can reach a single molecule; (4) it can satisfy the requirements of portable and bench-based testing environments.

**Fig. 11:**
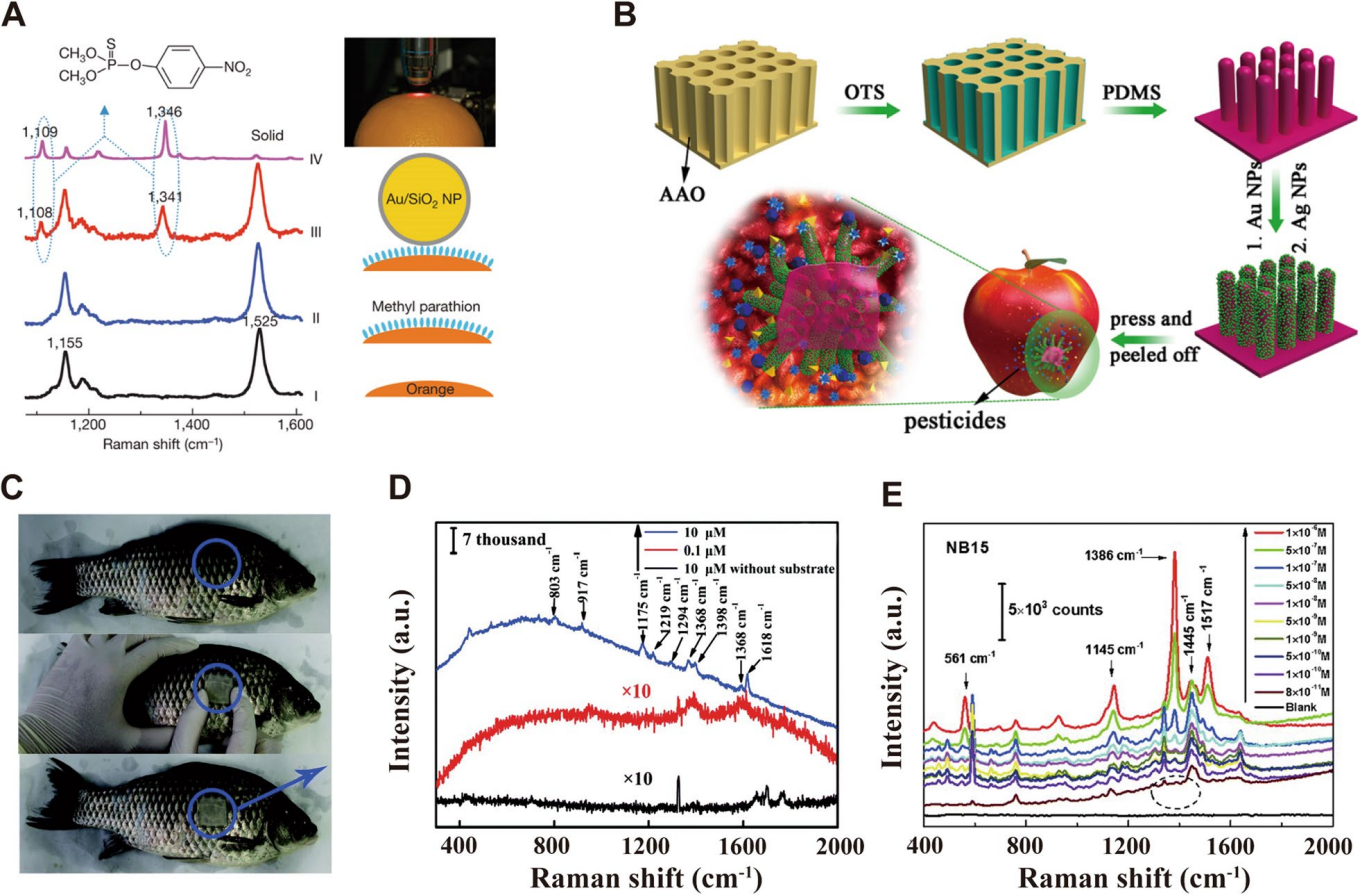
In-situ inspection of pesticide residues on food. **A** Schematic of the SERS experiment (right) and the corresponding Raman spectra on fresh citrus fruits (left). Spectrum I, with clean pericarps; spectrum II, contaminated by parathion. Spectrum III, spectrum of contaminated orange modified by Au/SiO_2_ nanoparticles. Spectrum IV, Raman spectrum of solid methyl parathion. [12] **B** Schematic demonstration of preparation of SERS substrate and SERS Measurement. [225] On-site detection of MG on **C** a living fish scale and corresponding **D** Raman spectra. [229] **E** The sensitivity to various concentrations of thriam based on Au@Ag nanocuboids. [231] **A** reprinted with permission from Ref. 12, © 2021, Springer Nature. Figure **B** reprinted with permission from Ref. 225, © 2017, American Chemical Society. **C** and **D** reprinted with permission from Ref. 229, © 2018, Royal Society of Chemistry. **E** reprinted with permission from Ref. 231, © 2015, Royal Society of Chemistry

In-situ detection is an outstanding advantage of SERS method for detecting pesticide residues. It does not require complex sample pretreatment process and allows direct detection on different sample surfaces. Usually, this method requires some delicate structure design of SERS substrates. Tian et al. reported a shell-isolated nanoparticles whose monolayers are scattered over the surface to be detected in the form of “smart dust” [12]. This method can be used for the detection of pesticide residues in food and fruits. As shown in Fig. 11A, the normal Raman spectra recorded on fresh oranges with clean peel (spectrum I) or fresh oranges contaminated with parathion (spectrum II) only show signals of citrus carotenoid molecules at 1155 cm^−1^ and 1525 cm^−1^. By dispersing the shell-isolated nanoparticles on the same surface, the characteristic bands of parathion residues at 1108 cm^−1^ and 1341 cm^−1^ (spectrum III) can be clearly detected. The shell-isolated structure prevents "smart dust" from caking and protects SERS active nanostructures from directly contact with detected objects, and allows "smart dust" detecting in-situ on different sample contours. Using the SERS method to detect pesticide residues in beverages, fruits or vegetables has been widely reported [222–228]. Rapid and non-destructive in-situ detecting is particularly important in food inspections. Organophosphate, pyrethroid and neonicotinoid are three kinds of insecticides commonly used in tea, fruit and vegetable cultivation. Hou et al. established an in-situ SERS method for detecting and identifying four pesticides (including two organophosphates (isocarbophos and phorate), a pyrethroid (deltamethrin) and a neonicotinoid (imidacloprid)) on plant surfaces without pretreatment [223]. This method only needs to drop AuNPs sol on the plant surface and the sol can be tested directly after drying with the LOD of 0.01 ppm and the total analysis time of 20 min.

Rapid detecting and the ability to satisfy the requirements of various test environments is another advantage of SERS technology. Due to the flexibility of available substrates, SERS technology can design suitable SERS substrates for different test scenarios. In order to protect fruits or vegetables from diseases and insect pests, different pesticides such as insecticides and fungicides are usually mixed, resulting in a variety of toxic pesticide residues. As shown in Fig. 11B, Wang et al. imitated the tentacles of the gecko designing a flexible nano-tentacle array (G-SERS), which only takes a few seconds by “press and peel” to complete the detection of pesticide residues on fruits surface with the LOD of 1.6 ng/cm^2^ [225]. Compared with ordinary SERS substrates, G-SERS substrates can achieve more efficient target sampling. It can be used to collect targets quickly and effectively from complex surfaces and simultaneously detect a variety of pesticide residues in real samples without any incubation. By directly sampling from the surface of cucumber, apple and grape, many components such as thiram, methyl parathion and malachite green (MG) can be determined quickly and reliably. In general, the traditional gold/silver sol–gel nanoparticle has excellent SERS performance, but the sol–gel is not suitable for long-term storage, have disadvantages such as easy oxidation and agglomeration. The functionalized SERS substrate like G-SERS substrates that are easy to carry, store and use is an important research direction for the practical application of SERS detection. Wei et al. developed a reusable Ag functionalized SERS substrate for the detection of MG residues on the scale surface of crucian carp [229]. As shown in Fig. 11C, D, 0.1 $\mu M$ concentration of MG residue can be detected by simply adhering and peeling the SERS substrate from the surface of fish scales within a few seconds. In addition to the advantages of rapid detection, SERS technology can also use portable devices for on-site rapid testing. Deng et al. can use portable Raman spectrometer to quickly detect MG residues in fish within 10 s, with LOD as low as 5 × 10^–10^ $M$ [230].

High sensitivity is also a major feature of SERS technology. The maximum residue limit (MRL) for pesticides in food set by the U.S. Environmental Protection Agency (EPA) is 7 ppm [231]. In addition, according to the national standard “*Determination of Malachite Green and Crystal Violet residues in Aquatic products*” issued by AQSIQ and National Standards Commission (GB/T19857-2005), the detection limits of Liquid Chromatography-Tandem Mass Spectrometry and High Performance Liquid Chromatography are 0.5 ppb and 2 ppb, respectively. In the actual detection using SERS method, LOD can easily reach the level of ppm or ppb [222, 223, 230–232]. Alsammarraie et al. developed a SERS substrate with AuNR arrays to detect and quantify carbaryl in orange juice, grapefruit juice and milk within 10 min with the LOD of 50 ppb [222]. In this study, the LOD of different beverages conformed to the MRLs of carbaryl, and the recovery of carbaryl extracted from actual food samples was also satisfactory. Guo et al. systematically investigated the size- and shape-dependent SERS activities of plasmonic core–shell nanoparticles towards detection of the pesticide Thiram [231]. Monodisperse Au@Ag nano-cubes (NCs) and Au@Ag nanocuboids (NBs) were synthesized. The LOD was 100 pM and 80 pM for NCs and NBs, respectively (Fig. 11E). In fact, the sensitivity of SERS substrate is only one of the aspects that we should consider, and the stability of substrate is also very important for practical application. Hu et al. designed a temperature-responsive poly (N-isopropyl acrylamide) (pNIPAM) AuNRs for detecting MG residues as low as 0.73 ppb in fish [232]. The SERS efficiency can be adjusted by temperature-induced expansion and collapse of nano-hybrids. Stable and uniform SERS signal was obtained on the substrate under continuous laser irradiation. The pNIPAM templates were separated from each other under the photothermal effect resulting in avoiding aggregation and still had high SERS performance after 3 months of storage. In Table 5, a summary of the above-mentioned schemes for the detection of pesticide residues in foods based on the SERS technique is presented. SERS-based method has higher sensitivity and selectivity to target analytes than traditional chromatographic methods and has great application potential in the identification of trace pesticide derivatives during food processing.

**Table 5:** Pesticide Residues in Different Food Matrices Detected by SERS

Pesticide	Matrix	Substrate	LOD	Refs.
Parathion	Orange	Au@SiO_2_	N/A	[12]
Isocarbophos/Phorate, Deltamethrin/Imidacloprid	Tea leaf/Apple peel	AuNPs	10 ppb	[223]
Thiram/Methyl parathion/Malachite green	Cucumber/Apple/Grape peel	Flexible nano-tentacle array	1.6 ng/cm^2^	[225]
Thiram/Malachite green	Fish	AgNP@AgNW	0.01 nM	[229]
Malachite green	Fish	AgNPs	0.5 nM	[230]
Carbaryl	Orange and Grapefruit juice/Milk	AuNRs	50 ppb	[222]
Thiram	N/A	Au@Ag nanocubes	80 pM	[231]
Malachite green	Fish	AuNRs	0.73 ppb	[232]

The sensitivity of SERS technology for the detecting pesticide residues is comparable to that of standard chromatography, while it has great advantages such as high speed, good flexibility, and can be used for in-situ detection in different scenes. Of course, there are still some obstacles to the large-scale application of SERS in food testing. The most important point is how to accurately pick up the signal we need in all kinds of interference signals on the food surface. The multivariate analysis method to extract information about multicomponent SERS spectra is a promising aspect. It is necessary to design a suitable SERS substrate for different kinds of pesticide detection and establish a standard database to accurately extract and analyze pesticide signals.

## Spectral recognition

The identification of biological Raman spectra can mostly be attributed to the spectral identification of certain proteins or amino acids. Therefore, the recognition and determination of biological spectra are inseparable from the resolution of protein spectra. In addition to chemical and structural analysis, protein detection provides theoretical analysis support for disease surveillance, such as protein denaturation or cellular carcinogenesis. The precise analysis of protein structure based on Raman spectroscopy by a large number of researchers has greatly facilitated our research. Thomas et al. have done a detailed analysis of protein structures and chemical bonding vibrations based on studies of viruses [233–235]. Bandekar analyzed the amide vibration modes and molecule conformations of protein and amino acids in detail [236]. Pelton and McLean gave some spectroscopic methods for the analysis of protein secondary structures, including methods of Raman spectroscopy [237]. Based on the work of Thomas, Bandekar and Pelton et al., [233–237] we provided a partial summary of the Raman shifts corresponding to the vibrational modes of amino acids or chemical bonds in proteins. However, it should be noted that due to the secondary structure of protein and the difference in substrate, the corresponding Raman shift may move in the range of a dozen wavenumbers.

We first focused on the vibrational modes of amides, which are the most fundamental vibrational modes of all amino acids. The amides of proteins carry nine vibrational modes, which in descending order of frequency are called A, B and I-VII. Amide I-VII is the most frequently analyzed vibration mode. Raman shift ranges for amide I-VII bands are given in Table 6 [233, 235, 236]. The most valuable Raman bands for proteins analysis are amide I and amide III, which have obvious Raman signals under the excitation of visible light. Amide I and amide III bands are quite sensitive to the protein secondary structure in their precise Raman shifts and band shapes. Therefore, amide bands are often used as indicators of protein secondary structures, such as protein $\alpha$-helix, $\beta$-sheet and disordered structures. The amide I conformational sensitive bands of $\alpha$-helix and $\beta$-sheet mainly locate in the range of 1645–1655 cm^−1^ and 1660–1680 cm^−1^, and the amide III conformational sensitive bands of $\alpha$-helix and $\beta$-sheet mainly locate in the range of 1260–1310 cm^−1^ and 1230–1245 cm^−1^ for amide III [233, 238]. Because the polarizability of the conformational vibrations for some amino acids in the protein structure varies greatly, the vibrations of these groups are expected to produce higher intensity in the Raman spectra [234]. For example, there are obvious in-plane stretching of C=O and C–N involved in peptide. Therefore, the amide group located in the plane is expected to produce relatively high Raman intensity. In addition, the vibration of aromatic side-chain (phenylalanine, tyrosine, tryptophan) is also considered to be strong in Raman spectra. The stretching vibrations of C–C, C–N and C–O, especially the symmetrical shifts of side-chain skeletons or carboxylates, also lead to strong Raman intensity. While the bending and stretching modes of hydrogen like substituents (C–H, N–H, O–H) are usually weak in Raman spectra. However, due to the large number of such groups in proteins or lipids, the collective Raman intensity may be very high. However, due to the presence of a large number of these aliphatic and other non-aromatic side chain groups in proteins or lipids, signal accumulation in certain bands may lead to high collective Raman intensities (e.g., C–H stretching vibrations of a large number of aliphatic groups in the 2850–2950 cm^−1^ region). Moreover, some strong Raman signals are related to group vibration involving the shifts of heavy atoms (such as sulfur). Therefore, there are some strong Raman bands related to the C–S stretching mode of methionine and cysteine, the S–S stretching mode of cystine (505–510 cm^−1^), the S–H stretching mode of cysteine and the Zn–S stretching mode of zinc-metallothionein.

**Table 6:** Vibrational properties of each amide mode

Amide mode	Potential energy distribution (PED)	Raman shift (cm^−1^)
Amide I	CO s(83), CN s(15), CCN d(11)	1618–1741
Amide II	NH ib(49), CN s(33), CO ib(12), CCs(10), NC s(9)	1509–1592
Amide III	NH ib(52), CCs(18), CN s(14), CO ib(11)	1226–1391
Amide IV	CO ib(44), CCs(34), CNC d(11)	627–800
Amide V	CN t(75), NH ob(38)	493–756
Amide VI	CO ob(85), CN t(13)	600–655
Amide VII	NH ob(64), CN t(15), CO ob(12)	202–226

*s* stretch; *d* deformation; *t* twist; *ib* in-plane bend, *ob* out-plane bend

In academic research, researchers will perform precise analysis of Raman spectra for biological molecules. However, in practical applications, it is usually necessary to rely only on some standard spectra to qualitatively determine the attribution of material signals. Therefore, rapid recognition based on feature spectra is crucial in practical applications. Based on the review of in situ Raman biosensing in the previous section, we observed that there are some obstacles in identifying characteristic spectra of biomacromolecule in clinical physiological settings due to the complex instinctive characteristic spectra of biomolecules and the sensitivity of the signal to the background [239]. Complex components of cells and similar structural composition of cytomembrane, cytoplasmic matrix, DNA, RNA, etc. lead to overlapping of characteristic peaks and giant instability for macromolecules’ Raman spectra. Fortunately, the combination between SERS technology and machine learning or deep learning with big data analysis ability provides a new pathway to analysis SERS spectra. Some mathematical statistics analysis methods, such as simple Principal Component Analysis (PCA), Discriminant Analysis (DA), Cluster Analysis, and some complex Decision Tree, Random Forest, Artificial Neural Network (ANN), Bayesian Learning, Support Vector Machine (SVM), could extract characteristic of complicate biomolecules’ Raman spectra and classify spectra with high accuracy [10, 240–242]. Taking advantage of above analytical technology, the practical application of SERS spectra could be developed to a new stage.

Feature extraction of complex spectra can be achieved using some simple machine learning methods such as PCA, DA, etc. The extracted feature vectors can be further combined with SVM or ANN to identify the spectra, which reduces the amount of data input to the latter and thus improves the data processing efficiency. Xu et al. used PCA to extract the feature variables of Raman spectra. The first four components of PCA were selected as the input values for the SVM classification model, which allowed for the rapid identification of sulfadimidine and sulfapyridine residues in duck meat [241]. The calculated sensitivity and specificity of the test set were 96.97% and 100%, respectively. Liu et al. also used SVM to identify Raman spectra of human serum samples to identify prostate cancer with higher accuracy than the results of PCA classification [240]. The effect of high Raman background on the signal can also be removed by some multivariate data processing means. Ye et al. used two multivariate curve resolution methods including negative matrix factorization and classical least squares to greatly mitigate the interference of autofluorescence signals from biological tissues or fluctuations caused by local states of nanoparticles (e.g. aggregation) [8, 243]. This method reduces the detection limit by an order of magnitude when detected against a high Raman background and minimizes the effect of fluorescence background and partially overlapping specific peaks during SERS imaging. However, many current spectral processing methods, such as regression analysis, discriminant analysis or SVM, also have some limitations. Most discriminant analysis relies on well-defined categories to train the model, requiring both information about all the spectra to be identified. For example, when identifying additives in food, the wide variety of additives hinders the modeling of all categories to train such models. In addition, new additives are constantly emerging and the models quickly become obsolete. As an alternative to deal with those problems, one-class classifiers are strongly recommended for authentication. These classifiers use only one well-defined class to train the model, sparing the need for other classification samples [244]. In the prediction step, if a sample does not belong to the unique class modeled, it will be set as the second class. Cardoso et al. proposed a new approach using Raman spectroscopy in tandem with one-class modelling SVM (OC-SVM) to meet this demand [245]. Although the Raman spectra of foods before and after the use of additives were highly similar, the OC-SVM method was able to achieve 87.1% and 86.8% sensitivity and specificity. Compared to classical linear spectral analysis methods, ANN is able to detect nonlinear dependencies, which are more suitable for complex (biological) samples that do not obey linear laws. Lyutakov et al. used ANN models to train SERS spectra of DNA to identify DNA damage with an accuracy higher than 85% [246]. Similarly, Zhao et al. modified DNA probes on the surface of AgNRs to capture RNA of SARS-CoV-2 and used recurrent neural network (RNN) models to identify RNA spectra [247]. The RNN model could predict 97.2% and 100% accuracy for positive and negative samples, respectively. Of course, the disadvantage of these fully connected neural network is that it has too many weights, which is computationally intensive and prone to overfitting. Huang et al. used a CNN model to train the Raman spectra of SARS-CoV-2, which avoids excessive computational effort despite the large amount of data on the input side. This CNN model can achieve 87.7% accuracy in identifying SARS-CoV-2 saliva samples without isolation or purification steps [109]. These results demonstrate that SERS platforms incorporating machine learning or deep learning algorithms greatly facilitate the application of SERS in biosensing.

Machine learning, as a discipline that intersects with many fields, has some application difficulties for some researchers who have just encountered this field. However, some open-source software or program package developed by researchers greatly facilitates the application of machine learning in the field of SERS. Wang et al. developed a kind of Raman spectra analysis software (NWUSA), which integrates spectra processing, analysis, and recognition of Raman characteristics [248]. It is an open-source software suitable for beginners for spectral processing and multivariate analysis, which provides a user-friendly graphical interface, pretreatment of executable spectra, and multivariate analysis algorithm including PCA, Linear Discriminant Analysis (LDA), Partial Least Squares discriminant analysis (PLS-DA) and SVM, etc. Wang et al. realized the different spectra recognition and analysis of ductal carcinoma (DCIS) and invasive ductal carcinoma (IDC) [249]. The software is easy to operate, but it also needs further optimization, such as poor compatibility, single data output form and so on. A simple, easy-to-use, fast and effective SVM pattern recognition and regression software package (LIBSVM) has been developed by Professor Lin Chih-Jen of Taiwan University [250]. It not only provides compiled executable files that could be used in windows systems, but also includes source code to facilitate improvement, modification and application in other operating systems. The software can solve C-SVM ν-SVM, ε-SVR, ν-SVR and other problems, including multi-pattern recognition based on one-to-one algorithm. Moreover, LIBSVM has dozens of language versions such as C, Java, MATLAB, C#, Ruby, Python, R, Perl, Common LISP, LabVIEW and PHP, which greatly facilitates its usage. Alternatively, there is also a quick start path to deep learning. The Fast Artificial Neural Network (FANN) library is a free open-source neural network library that implements multilayer artificial neural networks in C, supporting both fully connected and sparsely connected networks. It is easy to use and versatile, supporting multiple languages or platforms including Python, PHP, C +  + ,.NET, Delphi, Matlab, Octave, Ruby, Pure Data, Mathematica, etc.

## Challenges and perspectives

With decades of development, SERS sensing technology has been accepted by more and more researchers because of its rapid detection and high sensitivity. In the field of biosensors and medical diagnosis, SERS sensing has made tremendous progress, and some products have marched toward practicality. As discussed in previous sections, it has achieved various unprecedented experimental analysis and applications. Nevertheless, some challenges are required to be overcome for further promoting its development, especially the application of POC detection.

Firstly, more stable and repeatable SERS nano-tags should be developed for the label-SERS. Generally, label-SERS not suitable for POC detection in outdoor high-temperature environment because of poor thermal stability of SERS tags [251]. Especially for nano sol commonly used for SERS enhancement, as the nanoparticles increase in size, the nano sol is not able to undergo long-term storage due to the agglomeration. Moreover, Au/Ag nano sol is sensitive to ambient temperature, certain ions, or pH values, which also limit the application of SERS-enhanced substrates. Therefore, surface modification or suitable storage conditions of these nano sol is necessary to enhance their stability. Polymer encapsulation through SiO_2_ or PEG etc. is helpful to solve the stability of SERS tags.

Secondly, the non-uniformity of the traditional “hot spot” of SERS will cause poor signal reproducibility, which is also a significant challenge in practical application. As far as we know, many SERS products with excellent performance have failed to come out of the laboratory because of reproducibility issues. This may be due to the intrinsic performance of some SERS-enhanced substrates, such as the difficulty of achieving uniform size of Au/Ag nanosol particles. On the other hand, it could be that the experimentalists did not pay attention to the standardization of the production process and the storage of the enhanced substrates mentioned above. We discovered that most of the SERS sensors based on noble metals currently used have faced this problem. Most of the existing noble metal sensors introduce "hot spots" through chemical methods, and various chemical reagents would also bring severe signal interference, which is one of the reasons for the poor signal reproducibility. Perhaps this problem might be inapparent when the concentration of analytes is high. While this problem is particularly prominent when the concentration of analytes decreases to a certain value. Therefore, excessive chemical reagents should be avoided in the preparation of materials. The Ag/black phosphorus nanocomposite designed by our team utilized photoreduction to introduce "hot spots", which completely avoids the use of chemical reagents [157]. The nanosheets have no background of other signals in the biological fingerprint area, and could accurately locate single molecules at the concentration of 10^–20^ M. In addition, with reference to the semiconductor manufacturing industry, large-scale, standardized manufacturing processes are important for the manufacture of stable SERS substrates.

Thirdly, the interference of environment background signals is also a problem that SERS sensing has to face during the detection of analytes. Compared with other chemical analysis methods, SERS usually has remarkable detection sensitivity for pure chemical molecules. However, once target molecules are analyzed in the actual samples, its sensitivity would be descended rapidly due to the interference of the background, especially for biomolecules that is accompanied by generous impurities in the physiological environment. Improving the anti-interference capability of SERS detection is key to bringing it to practical applications and helps improve its signal reproducibility. On the one hand, appropriate nanostructures can be designed according to the structural characteristics of analytes to specifically enhance molecular signals or capture target analytes as simply as possible. Labelling SERS combined with microfluidic platform or LFA strips can also improve its interference resistance well. On the other hand, using the advantages of machine learning in big data processing to identify spectra has become more and more important. Finally, the toxicity and stability of SERS probe need to be further evaluated and optimized for internal in-situ detection or cell imaging.

## Summary

Here, we reviewed the latest application progress of SERS biosensor in several common fields. Starting from the basic principle of SERS enhancement, we introduced the development process of SERS enhancement mechanism. Subsequently, we presented some surface modification approaches of SERS substrates most commonly used in the detection of biomacromolecules. Afterwards, we focused on the research and practical application of SERS sensors in different fields, including virus detection, tumor detection, biological imaging and drug detection. There are many excellent research results and advanced research ideas, which provide great convenience and guidance for our research work. Eventually, we pointed out some challenges in the development of SERS biosensors, and put forward the corresponding solutions. The value of SERS technology in the field of biosensor is worthy to be affirmed, and it is moving towards practicality for more. The maturation of microfluidic chip, LFA platform and machine learning technology also assist the application of SERS sensing in POC test. We believe that more and more SERS products that could be really applied to biochemical molecular sensing and disease diagnosis will appear in the future.

## Data Availability

Not applicable.
